# TaqTth-hpRNA: a novel compact RNA-targeting tool for specific silencing of pathogenic mRNA

**DOI:** 10.1186/s13059-024-03326-3

**Published:** 2024-07-07

**Authors:** Chong Xu, Jiyanuo Cao, Huanran Qiang, Yu Liu, Jialin Wu, Qiudan Luo, Meng Wan, Yujie Wang, Peiliang Wang, Qian Cheng, Guohua Zhou, Jian Sima, Yongjian Guo, Shu Xu

**Affiliations:** 1 School of Basic Medical Science and Clinical Pharmacy, China Pharmaceutical University, Nanjing, 210009 China; 2 Laboratory of Aging Neuroscience and Neuropharmacology, China Pharmaceutical University, Nanjing, 210009 China; 3 Department of Pharmacology, Jinling Hospital, Medical School, Nanjing University, Nanjing, 210008 China; 4 School of Biopharmacy, China Pharmaceutical University, Nanjing, 210009 China

**Keywords:** TaqTth, Hairpin RNA guiding probe, RNA cleaving, Alzheimer’s disease, AAV, APP, APOE2

## Abstract

Pathogenic allele silencing is a promising treatment for genetic hereditary diseases. Here, we develop an RNA-cleaving tool, TaqTth-hpRNA, consisting of a small, chimeric TaqTth, and a hairpin RNA guiding probe. With a minimal flanking sequence-motif requirement, in vitro and in vivo studies show TaqTth-hpRNA cleaves RNA efficiently and specifically. In an Alzheimer’s disease model, we demonstrate silencing of mutant *APP*^*swe*^ mRNA without altering the wild-type *APP* mRNA. Notably, due to the compact size of TaqTth, we are able to combine with APOE2 overexpression in a single AAV vector, which results in stronger inhibition of pathologies.

**Supplementary Information:**

The online version contains supplementary material available at 10.1186/s13059-024-03326-3.

## Background

The discovery of diverse RNA-targeting strategies enables efficient RNA editing, RNA virus inhibition, nucleic-acid detection, and of course gene therapy. Gene therapy, especially mediated by post-transcriptional RNA knockdown, has the potential to be an ideal approach for the treatment of diseases caused by pathogenic mutations [1]. For example, in familial Alzheimer’s disease (FAD), the allele-specific silencing of pathogenic APP Swedish mutation (*APP*^*swe*^) [2], which results in excess Aβ production [3, 4], is a valuable weapon in the therapeutic arsenal against FAD. However, given that wild-type APP plays crucial physiological roles in CNS development, off-targeted altering of the amounts of wild-type APP (only one or two bases difference with mutant pathogenic APP in coding sequence) is probably to worsen AD symptoms [5]. Hence, more high-specific RNA-knockdown tools should be explored to discriminate minimal base differences on targets.

Previously, we have reported that the flap endonuclease 1 (FEN1) can be guided by DNA probes, but not RNA probes, to cleave targeted RNA [6]. Although it could distinguish single-base differences on the “seed region” of targeted substrates, the efficiency should be further improved. Meanwhile, diverse robust RNA-cleaving CRISPR effectors have been developed, which have the common character of being RNA-guided. Although the sgRNAs were transfected into cells as the DNA type of plasmids or PCR products [7], a big amount of transcription production contributes significantly to the CRISPR system’s high efficiency. Compared with abundant sgRNAs, the amount of DNA probes spread freely into the nucleus is finite. It inspires us to explore high-specific RNA nucleases guided by RNA probes.

As reported [8, 9], the *Thermus aquaticus* and *Thermus thermophilus* DNA polymerases (shorted as TaqPol and TthPol, respectively) have been reported to possess RNA-dependent 5’ nuclease activity. Besides, the polymerase family contains very similar functional domains as FEN1 [10, 11], inferring that polymerase may also be sensitive to mismatches on targeted substrates just as FEN1 [12]. It may also be with the advantage of minimal sequence-motif requirements like the FEN1-associated system [6, 13]. Thus, we speculate that the polymerase can recognize RNA probes and could be further engineered as a high-efficient RNA-cleaving tool.

In this study, the novel RNA-cleaving tool will be constructed and tested in vitro and in vivo, including efficiency, specificity, sequence-motif requirements, and cell toxicity. Then we will apply it to demonstrate a strong knockdown against *APP*^*swe*^ mutation, avoiding altering the wild-type *APP* mRNA. Considering the compact size of TaqTth, the combination of it and human apolipoprotein E 2 (APOE2) overexpression encoded in a single AAV vector will be tried to inhibit the pathologies in the mouse model. We thus believe that this novel toolkit, as an alternative to now available systems, may have the potential to provide precious tools for gene therapy strategies.

## Results

### The design of the compact TaqTth-mediated RNA-cleaving tool TaqTth-hpRNA

**Fig. 1:**
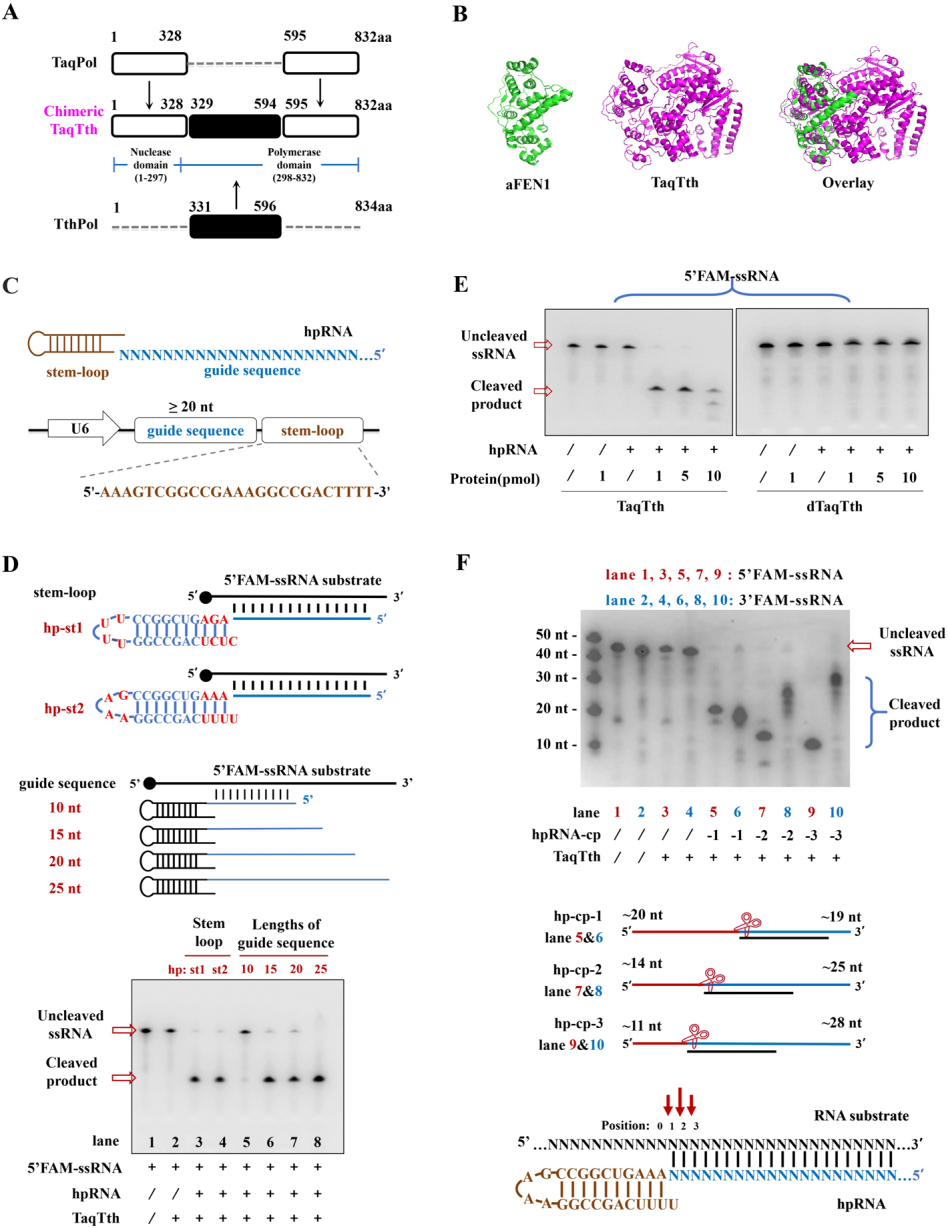
The design of the TaqTth-hpRNA and validation in gel-experiments. **A** The chimeric TaqTth protein contains two domains from TaqPol (1-328; 595-832 aa, hollow square) and one domain from TthPol (331-596 aa, solid square). **B** Phyre 2 simulation 3D structure based on the chimeric TaqTth. The N-terminal of TaqTth structure is similar to *A. fulgidus* FEN1. **C** The design of hpRNA, containing a stem-loop sequence (the brown part) at the 3' terminal and a guide sequence (the blue part) at the 5' terminal. **D** Denaturing gels showing TaqTth catalyzed cleavage of targeted 5’FAM-ssRNA guided by different types of stem-loop hpRNAs (hp-st1 and hp-st2). The effect of guide sequences with different lengths ranging from 25 to 10-nt at an interval of 5-nt. **E** Denaturing gels showing the loss of RNA-nuclease activity of dTaqTth (D144A, K418A, and Q507A). **F** Denaturing gels showing bands of cleaved products shifting with different hpRNAs (hp-cp-1, hp-cp-2, hp-cp-3) and the possible cutting positions on the targeted ssRNA

First, of the various enzymes in the polymerase family, a chimeric TaqTth protein (Fig. 1A, 832 amino acids), containing two domains from TaqPol and one domain from TthPol, was reported to have higher RNA-dependent 5’ nuclease activity than others [8]. It was reported that the 1–328 amino of TaqPol contains a high specific 5’ nuclease domain, and the 331–596 amino of TthPol determines its high ribonuclease activity. The rearrangement of these crucial structural domains enables the chimeric TaqTth to possess high efficiency and specificity. A homology modeling by Phyre 2 was performed to predict the chimeric TaqTth structure (Fig. 1B, the purple) and was further compared with *A. fulgidus* FEN1 protein (aFEN1) by PyMol (Fig. 1B, the green) based on characterized structural homologs. Upon examination, we noticed the structures of aFEN1 and the N-terminal of TaqTth were partly overlapped, inferring that it may also be sensitive to mismatches on targeted substrates and with the advantage of minimal sequence-motif requirements, just as FEN1 [12]. Hence, the TaqTth was picked up as the nuclease component of our RNA-cleaving tool.

Next, a hairpin RNA guiding probe (shorted as hpRNA) was designed by imitating the hairpin DNA guiding probe (shorted as hpDNA) from the previously reported HpSGN [6], since the TaqTth has a similar domain as aFEN1. As shown in detail in Fig. 1C, the hpRNA contains a stem-loop sequence (the brown part) at the 3' terminal and a guide sequence (the blue part) at the 5' terminal. Originally, we designed the stem-loop sequence (hp-st1 in Fig. 1D) identically to that in the hpDNA [6]. Considering the hpRNA is mediated by the U6 promoter in cells, we advanced the stem-loop sequence (hp-st2 in Fig. 1D) to make the transcription stop at the end of the stem-loop but not terminate in the middle. Then an in vitro examination was carried out to determine whether the TaqTth protein can efficiently cleave targeted RNAs when guided by hpRNA or not. The targeted single-stranded RNA (ssRNA) was synthesized with FAM modification in its 5’ end, associated with their in vitro artificially synthesized hpRNAs (Additional file 2: Table S1). Theoretically, only the intact ssRNA (uncleaved) and the cleaved products containing the 5’ end of targets with the FAM can be visibly observed. Indeed, both the hp-st1 and hp-st2 guided the purified TaqTth to successfully catalyze the cleavage of the ssRNA target (Fig. 1D). Subsequently, the length assay of the guide sequence in hpRNA was implemented. Guide sequences of 10-, 15-, 20-, and 25-nt length were used (Additional file 2: Table S1), respectively. Even though the 10-nt guide sequence did not work, the 15-nt, 20-nt, and 25-nt guide sequences performed well (Fig. 1D). We assume that the guide sequence should be long enough to bind firmly on the substrate. Hence, a more than 20-nt guide sequence and the hp-st2 version of stem-loop were employed in the hpRNA (Fig. 1C). The molecular interaction between TaqTth and hpRNA was simulated by AlphaFold (Additional file 1: Fig. S1A).

According to the literature [8, 10, 14], the activity of the chimeric TaqTth is likely influenced by the D144, K418, and Q507 sites. The “dead” mutated dTaqTth protein was obtained by mutating these three sites to alanine. The dTaqTth was incapable of cleavage against RNA substrates (Fig. 1E) while remaining binding ability (Additional file 1: Fig. S1B). It could be utilized as a control in the next cellar tests.

To further identify the cutting sites, the 5' end and 3' end of targeted ssRNA were labeled with FAM, respectively, and three hpRNAs (hp-cp-1, -2, -3) shifted from 3' to 5' in sequence (Fig. 1F, Additional file 2: Table S1). It was observed that the cleavage products of the 5' end labeled ssRNA decreased from large to small (about 20-nt, 14-nt, 11-nt in sequence), while the cleavage products of the 3' end-labeled ssRNA increased from small to large (about 19-nt, 25-nt, 28-nt in sequence). We inferred that the cutting site falls near the 5' end of the probe-target duplexes region (Fig. 1F).

As a result, we collaborated with the chimeric TaqTth and appropriate hpRNAs to create a novel RNA cleavage system and named it as TaqTth-hpRNA.

### The cleaving of mammalian transcripts by the TaqTth-hpRNA

**Fig. 2:**
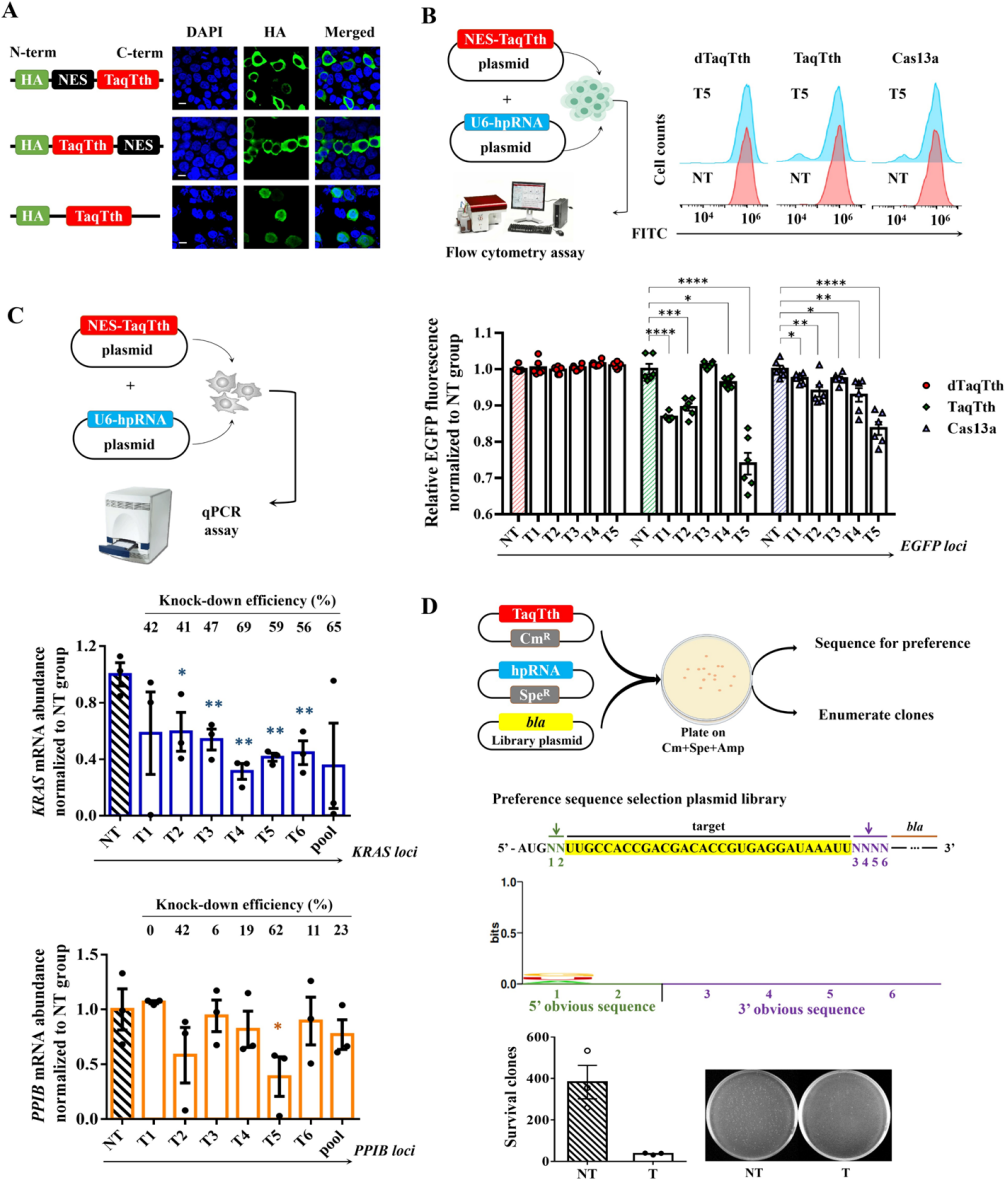
The feasibility of cleaving mRNA guided by TaqTth-hpRNA. **A** Engineering of the TaqTth with NES enables its expression in the HEK293T cell cytoplasm. HA represents the TaqTth expression. Scar bar = 10 μm. **B** The relative fluorescence and the distribution of EGFP-positive/negative cells in groups treated by TaqTth, dTaqTth, and Cas13a on different loci. mean ± s.e.m. *n* = 6. *p < 0.05, **p < 0.01, ***p < 0.001, ****p < 0.0001. *T1* to *T5* mean 5 different loci on the *EGFP* mRNA. *NT* means the non-targeting. **C** The schematic diagram process of the endogenous mRNA assay. The hpRNA-guided knockdown of *KRAS* and *PPIB* mRNA on different loci. mean ± s.e.m. *n* = 3. *p < 0.05, **p < 0.01. *T1* to *T6* mean 6 different loci on the targeted mRNA. *pool* means the mixture of those targeting hpRNAs. *NT* means the non-targeting. **D** Schematic of bacterial assay for determining the sequence preference of TaqTth. Bacterial plasmids expressing TaqTth and hpRNA are co-transformed with bla-plasmids containing randomized sequence preferences and subjected to triple antibiotic selection. The clones that are depleted during co-transformation with TaqTth suggest targeting activity and are used to infer sequence preferences. Preferences are derived from sequences depleted in the TaqTth condition relative to NT controls

To verify whether the TaqTth-hpRNA can be reprogrammed to work in mammalian cells, the hpRNA probe was inserted following a U6-promoter to form a pU6-target plasmid (Fig. 1C, Additional file 1: Fig. S2) and the TaqTth encoding gene (2496 bp + *TGA*, 832 amino acids, Additional file 1: Fig. S3) was inserted into pcDNA3.1 with nuclear export sequence (NES) attached at N- or C-terminal. It restricted TaqTth expression in the cytoplasm (Fig. 2A, Additional file 1: Fig. S4); otherwise, nuclear expression of the TaqTth may inevitably change the genomic DNA and increase the off-target effect. Plasmid individually expressing NES-TaqTth (the NES was attached at the N-terminal of TaqTth) or TaqTth-NES (the NES was attached at the C-terminal of TaqTth) was co-transfected with pU6-hp-EGFP (Additional file 2: Table S2) into HepG2 cells containing EGFP plasmids, followed by flow cytometry detection of the EGFP expression levels. In the NES-TaqTth group, the EGFP-positive cells decreased more than that in the NT (non-targeting) control group (Additional file 1: Fig. S5, from 22.7% to 14.8%). In contrast, no similar decline was observed in the TaqTth-NES group (Additional file 1: Fig. S5, from 18.5 to 19.3%). Therefore, the NES-TaqTth was used in the following study.

To further explore the TaqTth-hpRNA in mammalian cells, we detect several targets located at different loci (Additional file 2: Table S2) with an EGFP stable transfected HEK293T cell line. As shown in Fig. 2B and Additional file 1: Fig. S6, the knockdown efficiencies of EGFP expression levels in the groups with hp-EGFP were ~ 29.5% at the best level. The comparison with Cas13a demonstrated their similar efficiency. Then we tested whether our TaqTth-hpRNA tool works for endogenous mRNA. The HepG2 cells were co-transfected with plasmids encoding NES-TaqTth and pU6-target for *KRAS* mRNA (Additional file 2: Table S2), followed by detection of the *KRAS* mRNA levels (Additional file 2: Table S3). The qPCR data showed that significant down-regulation of *KRAS* mRNA level (~ 68.7% at the best level, Fig. 2C, Additional file 1: Fig. S7) was observed. In addition, the experiments of repression of *PPIB* mRNA, another endogenous target, were also conducted and a similar knockdown efficacy was achieved (~ 61.3% at best level, Fig. 2C). These data showed that the TaqTth-hpRNA tool can effectively cleave endogenous transcripts in mammalian cells. We also found that it is somewhat analogous to the varying cleavage efficiencies of siRNA technology on different targets, whereby different secondary structures of targets may possess varying binding capabilities with hpRNAs.

In the above gel or cell experiments, targeted loci were chosen randomly without sequence motif requirements like PFS. To deeply clarify whether the potential preference of sequences is required for the TaqTth-hpRNA, referring to the CRISPR article [15], a plasmids-library for preference screens was designed by inserting two 5’ randomized nucleotides and four 3’ randomized nucleotides (corresponding to the position of PFS in CRISPR/Cas13) separated by a target site immediately downstream of the start codon of the ampicillin resistance gene *bla* (Fig. 2D). The TaqTth-hpRNA successfully broke the target and made parts of clones unable to surviving on ampicillin plates (Fig. 2D). To assess preferences, the positions containing randomized nucleotides in the TaqTth-hpRNA treated group were extracted and sequenced relative to the control group. No sequence preference was found in these six loci by sequencing analysis (Fig. 2D), which provides an attractive advantage.

### The cytotoxicity and specificity of TaqTth-hpRNA interference activity

**Fig. 3:**
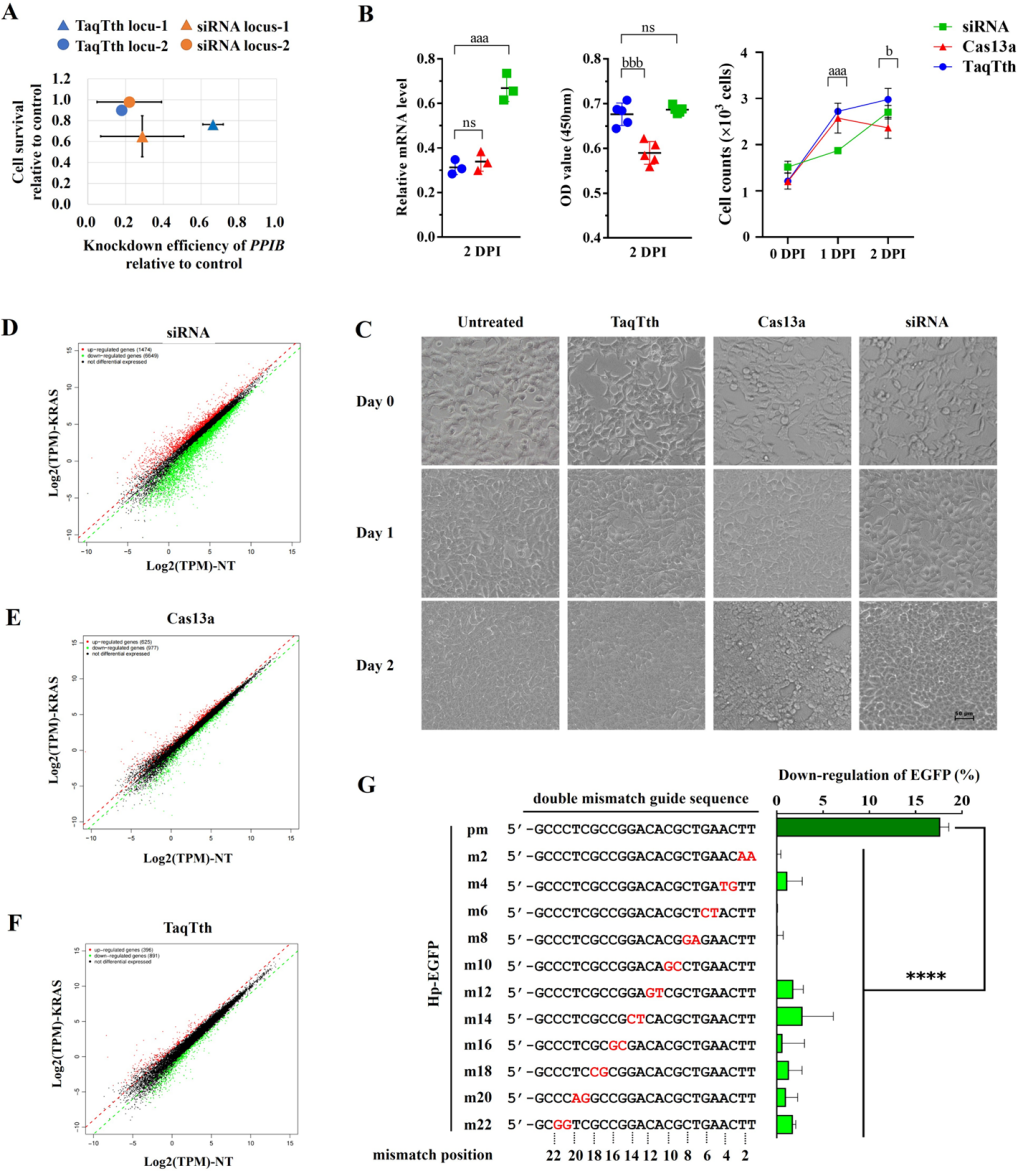
The cell toxicity and collateral effect of the TaqTth-hpRNA, Cas13a, and siRNA. **A** Cell viability of HepG2 cells during *PPIB* targeting by TaqTth and siRNA. For *x*-axis, mean ± s.e.m, for *y*-axis, mean ± s.d. *n* ≥ 3. **B** The knockdown efficiency, the optical density (OD) value in CCK8 assay, and the growth curve of different cells treated by TaqTth, Cas13a, and siRNA targeting the same *KRAS* mRNA locus. mean ± s.d. *n* = 3 or 5. ^aaa^*p* < 0.001 compared with siRNA group. ^bbb^*p* < 0.001, ^b^*p* < 0.05 compared with Cas13a group. *ns* means no significant difference. **C** Representative bright-field images of different cell clones during the 2 days after treated by TaqTth, Cas13a, and siRNA targeting the same *KRAS* mRNA locus. Scale bar = 50 μm. **D**,** E**, **F** Scatter plots of differential gene expression levels in RNA-seq between siRNA-mediated (**D**) Cas13a-mediated (**E**) and TaqTth-mediated (**F**) *KRAS* mRNA degradation in HEK293T cells. The significant up-regulated and down-regulated genes were marked in red and green dots, respectively. The values in *x*-axis and *y*-axis were the logarithm values of transcripts per million (TPM) in the non-targeting (NT) group and the targeting group, respectively. **G** Validation of TaqTth’s specificity to mismatches. Left: individual perfect matching (pm) and mismatch hpRNAs to *EGFP* targeted mRNA. Right: percentage of *EGFP* down-regulation normalized to the non-targeting control. mean ± s.e.m. *n* = 3. The mismatched bases were marked in red. ****p < 0.0001

The results above proved the capability of the TaqTth-hpRNA to cleave mammalian mRNA with two favorable features of small size and no sequence-motif requirement. To further investigate whether the TaqTth-hpRNA possibly causes more detectable cell toxicity, we compared it with the siRNA at the same loci (Additional file 2: Table S4). Data showed that the knockdown efficacy of endogenous *PPIB* mRNA and survival rates of cells treated by the TaqTth-hpRNA is better than that of siRNA (Fig. 3A). Comparation with Cas13a was also conducted. The hpRNA, siRNA, and gRNA were designed at the same loci (Additional file 2: Table S4). The parallel *KRAS* knockdown efficiency at 48 h after being treated by TaqTth and Cas13a was observed, which was better than that of siRNA (68.8%, 66.2%, and 33.2% relative to each control, respectively, Fig. 3B). We also looked into the cell survival and found that TaqTth-hpRNA and siRNA treatment had lower cell toxicity and a better cell state (Fig. 3B, C) compared to Cas13a.

To examine the intensity of the collateral effects of the TaqTth-hpRNA, we performed a total RNA integrity analysis of *KRAS*-knockdown HEK293T cells treated with TaqTth-hpRNA, Cas13a, or siRNA. Similar to previous findings [16], a significant reduction in RNA integrity was observed in the siRNA-treated cells (Additional file 1: Fig. S8). We also observed off-target degradation of transcripts in *KRAS*-knockdown HEK293T cells by transcriptome-wide RNA-seq. Data showed that ~ 1474 and 6649 genes were significantly up-regulated and down-regulated in the siRNA group compared with the NT control (Fig. 3D). Unlike siRNA treatment, the Cas13a and TaqTth-hpRNA sharply reduced the number of off-target genes when targeting *KRAS* (Fig. 3E, F), indicating that TaqTth mainly reduced collateral off-target degradation.

To further understand the sensitivity for mismatches, in the EGFP-stable cell line, a set of hpRNAs was designed to introduce double mismatches at different positions in the EGFP guide sequence (Fig. 3G) and used flow cytometric analysis to measure the efficiency of down-regulation for EGFP. Compared with the group of Hp-EGFP-pm (perfect matching to the targeted *EGFP* mRNA), the groups of mismatched Hp-EGFPs (Hp-EGFP-m2 to Hp-EGFP-m22) showed a much lower level of *EGFP* down-regulation (Fig. 3G). Moreover, this sensitivity for mismatches reflects obvious preference when placed at the 3’ terminal of the guide sequence. We also introduced single mismatches in Hp-EGFPs (Hp-EGFP-m1 to Hp-EGFP-m9), which resulted in about eight times reduction in the efficiency relative to that in the Hp-EGFP-pm group (Additional file 1: Fig. S9).

### High-specific targeting of pathogenic *APP*^*swe*^mRNA by TaqTth-hpRNA

**Fig. 4:**
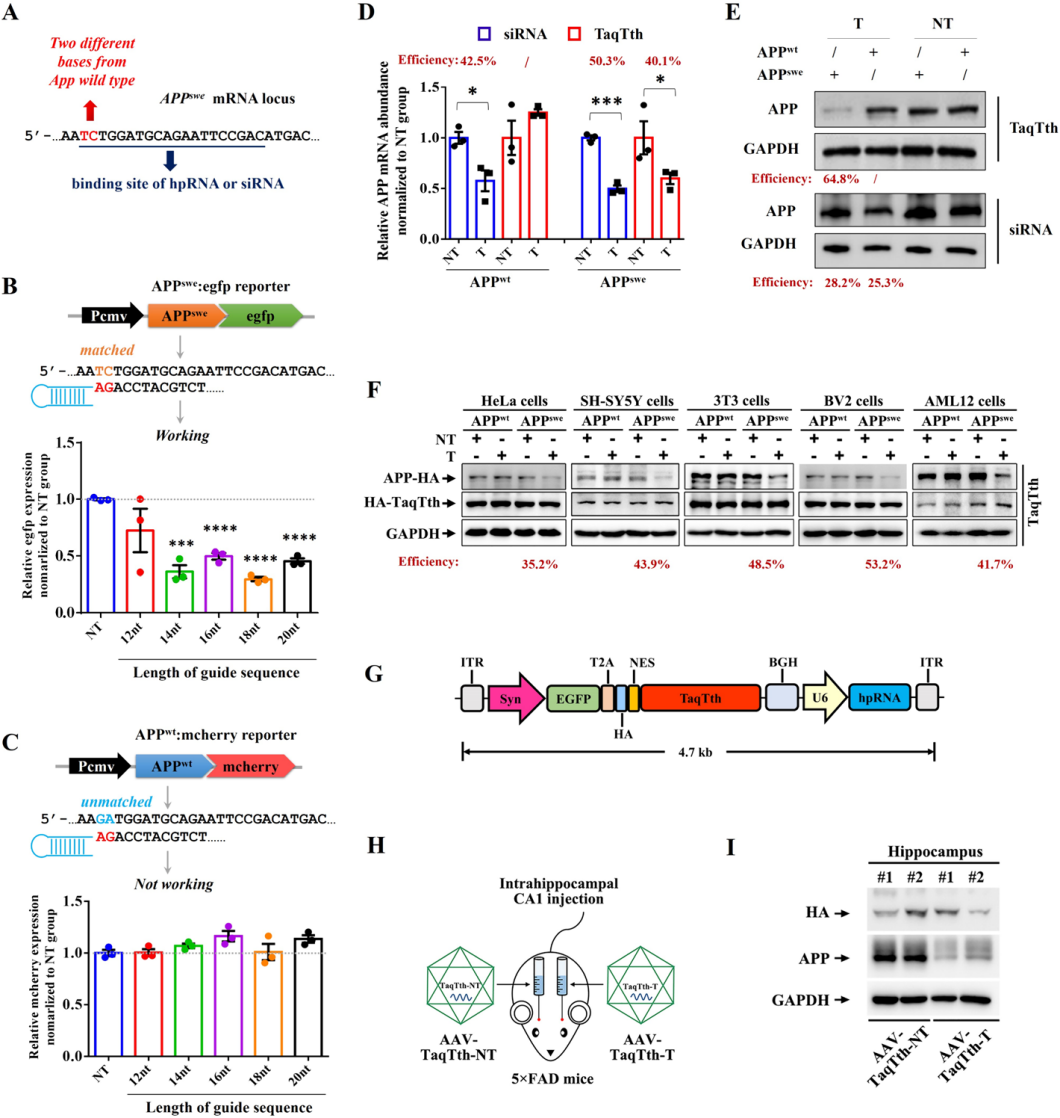
High-specific targeting of pathogenic *APP*^*swe*^ mRNA by TaqTth-hpRNA*. ***A** The design of the locating site of the hpRNA and siRNA on the mutant *APP*^*swe*^ mRNA. The mutant bases were marked in red. **B**,** C** The APP-fluorescence reporter assays to screen the suitable length of guide sequence in hpRNA. mean ± s.e.m. *n* = 3. ****p < 0.0001, ***p < 0.001. *NT* means the non-targeting hpRNA. **D** Relative *APP*^*swe*^ and *APP*^*wt*^ knockdown efficiencies on mRNA abundance level induced by TaqTth-hpRNA or siRNA. *p < 0.05, ***p < 0.001. mean ± s.e.m. *n* = 3. *NT* means the non-targeting hpRNA or siRNA. *T* means the targeting hpRNA or siRNA. **E** The silencing efficiencies of APP^swe^ and APP^wt^ on protein level induced by TaqTth-hpRNA or siRNA. *NT* means the non-targeting hpRNA or siRNA. *T* means the targeting hpRNA or siRNA. The efficiency was calculated by the grey value of bands (APP^swe^: lanes 1 and 3, APP^wt^: lanes 2 and 4, from left to right). **F** Replication experiments on the efficiency of TaqTth in targeting APP^swe^ across various cell lines. **G** A single AAV9 vector shuttle plasmid schematic. **H** Schematic shows intrahippocampal injection of AAV-TaqTth-NT and AAV-TaqTth-T particles. *NT* non-targeting, *T* targeting. **I** Immunoblotting shows the protein levels of HA and APP in the different hippocampal sides of 5 × FAD mice injected with AAV-TaqTth-NT and AAV-TaqTth-T. GAPDH as a loading control. *n* = 2 mice. HA represents the expression of TaqTth

To fully utilize the high specificity of TaqTth-hpRNA, we extended our study into disease-modifying research. AD has been tried to treat with more than 100 different substances thus far, but none of them have been successful in slowing the progression of the condition [17]. Most cases of familial Alzheimer’s disease are caused by heterozygous monogenic mutations of APP [2], among which the Swedish mutation (*APP*^*swe*^, K670N/M671L) is located at the cleavage site of β-secretase, resulting in stronger cleavage and excess Aβ production [3, 4]. Allele-specific RNA-based therapies may prove to be a valuable weapon in the therapeutic arsenal against AD. We intended to determine whether the TaqTth-hpRNA tool could specifically target *APP*^*swe*^ mRNA, while the *APP*^*swe*^ mRNA has only two mutated bases to the wild-type *APP* gene (*APP*^*wt*^) (Fig. 4A).

To avoid targeting the *APP*^*wt*^ mRNA, we placed these two different bases at the 3’ terminal of guide sequence of hpRNA (Fig. 4A, Additional file 2: Table S2) and screened the suitable length of guide-sequence in HepG2 cells expressing a reporter plasmid encoding *APP* mRNAs (the *APP*^*swe*^* or APP*^*wt*^ cDNA was fused in *egfp* or *mcherry* cDNA, respectively, Fig. 4B, C). As expected, the fluorescence intensity indicated the mRNA level of *APP*^*swe*^ or *APP*^*wt*^. The 12-bp length of the guide sequence showed low cleaving efficiency on *APP*^*swe*^ mRNA and cross-reaction on *APP*^*wt*^ mRNA. However, the other hpRNAs with 14-, 16-, 18-, and 20-bp length of guide sequence specifically knocked down the *APP*^*swe*^, but not *APP*^*wt*^ mRNA.

To further investigate the efficiency and specificity, we generated two HEK293T cell lines stably expressing APP^swe^ or APP^wt^ (HEK293T-APP^swe^/HEK293T-APP^wt^ cells), and detected the levels of APP in these cells by qPCR (Additional file 2: Table S3) and WB. The TaqTth-hpRNA with the 20-bp length guide sequence was examined by comparison with the siRNA, which had the identical sequence as the guide sequence in the hpRNA (Fig. 4A, Additional file 2: Table S4). For APP^swe^ knockdown in the HEK293T-APP^swe^ cells, in contrast to the NT control groups, remarkable down-regulation of *APP*^*swe*^ mRNA (~ 40.1% in TaqTth-hpRNA groups and ~ 50.3% in siRNA groups, respectively, Fig. 4D) was observed. In HEK293T-APP^wt^ cells, ~ 42.5% down-regulation of *APP*^*wt*^ mRNA was also noted by the treatment of the same siRNA, while the TaqTth-hpRNA treatment showed no detectable change on *APP*^*wt*^ mRNA (Fig. 4D). Levels of down-regulation in the protein aspect were also analyzed (Fig. 4E). Consistently, immunoblotting showed that TaqTth-hpRNA decreased the levels of APP^swe^ protein (~ 64.8%), but not APP^wt^ protein, indicating that TaqTth-hpRNA specifically targeted the *APP*^*swe*^ mRNA (Fig. 4E). Again, the siRNA showed lower knockdown efficiency on APP^swe^ (~ 28.2%), and like the mRNA levels, the protein levels of APP^wt^ were also down-regulated (~ 25.3%, Fig. 4E). In addition, we conducted replication experiments in various cell lines, including human cervical cancer HeLa cells, human SH-SY5Y neuroblastoma cells, mouse 3T3 fibroblast cells, mouse BV2 microglia cells, and mouse AML12 hepatocytes. We found that TaqTth-hpRNA effectively reduced the levels of APP^swe^ protein without affecting the APP^wt^ protein levels (Fig. 4F). Taken together, these data prove that our TaqTth-hpRNA has a better knockdown efficacy as well as a higher specificity than the siRNA used here and maybe a valuable approach to interfere with genetic disorders such as FAD.

Then we constructed a single AAV9-based vector separated expression EGFP and TaqTth mediated by the CAG promoter together with hpRNAs transcription driven by a U6 promoter (Additional file 1: Fig. S10). The knockdown efficiency was validated and data showed that the mRNA levels of *APP*^*swe*^ were markedly down-regulated by TaqTth-hpRNA (~ 40.2%, Additional file 1: Fig. S10), while no change was noticed either on *APP*^*wt*^ levels. Again, the protein levels of APP^swe^ were also clearly reduced by TaqTth-hpRNA (~ 41.5%) with no significant influence on APP^wt^ levels (Additional file 1: Fig. S10).

To validate the practicality of the TaqTth-hpRNA to silence the *APP*^*swe*^ in the mouse model of AD, the CAG promoter was replaced with the neuron-specific synapsin I (Syn) promoter (Fig. 4G), then produced AAV9 viral particles and named as AAV-TaqTth-NT (containing NT hpRNAs) and AAV-TaqTth-T (containing targeting hpRNAs), respectively. We then used stereotactic injection of AAV-TaqTth-NT or AAV-TaqTth-T into the hippocampi of 3-month-old mice of the 5 × FAD strain (Fig. 4H), which expresses mutant human APP (the Swedish mutation: K670N, M671L; the Florida mutation: I716V; the London mutation: V717I) and PS1 (M146L; L286V). One month later, hippocampal tissues were collected and immunoblotting showed that the protein levels of APP were reduced (~ 68.4%) by AAV-TaqTth-T compared to AAV-TaqTth-NT (Fig. 4I). Thus, our results prove that the TaqTth-hpRNA tool can specifically and efficiently knock down mutated APP in the brains of the 5 × FAD mouse.

### Silencing mutant APP by TaqTth-hpRNA reduces cognitive decline and alleviates AD pathologies in the 5 × FAD mice

**Fig. 5:**
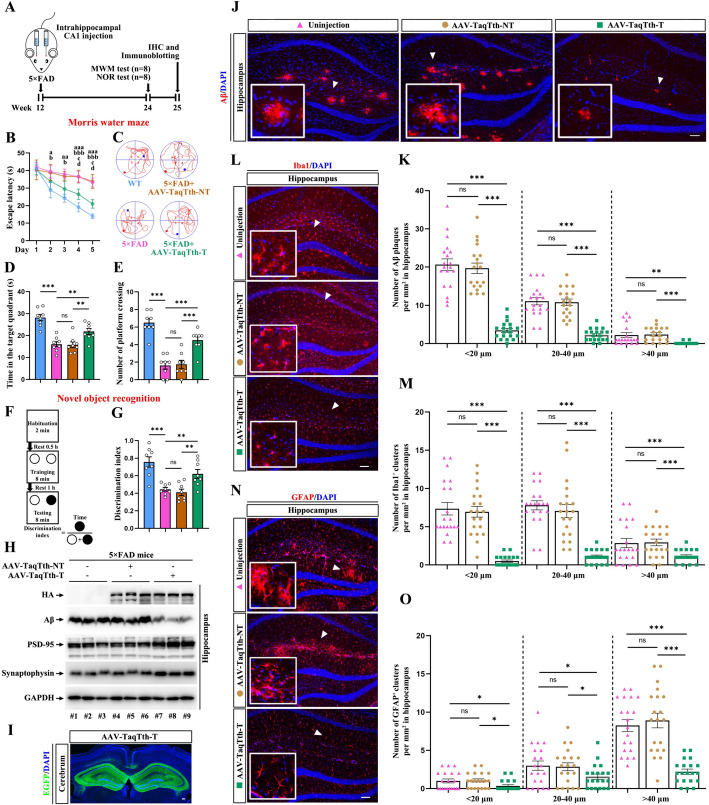
AAV-delivered TaqTth-hpRNA improves cognitive deficits and decreases Aβ pathologies in 5 × FAD mice. **A** Experimental setup. Three-month-old 5 × FAD mice underwent intrahippocampal injection of AAV-TaqTth-T or AAV-TaqTth-NT, followed by behavioral and pathological examinations at the age of 6-month-old in comparison with WT or 5 × FAD mice of the same age without injection. **B**,** C**,** D**,** E** Effect of AAV-TaqTth-T or AAV-TaqTth-NT on cognitive deficits in 5 × FAD mice assayed by MWM. The quantitation of escape latency during cued training (**B**). The representative trajectories (**C**), the time in the platform located quadrant (**D**), and the average number of platform crossings (**E**) in the probe trial. *n* = 8 mice. Data were analyzed by one-way or two-way ANOVA with Fisher’s LSD post hoc tests. mean ± s.e.m. ^a^*p* < 0.05, ^aa^*p* < 0.01, ^aaa^*p* < 0.001 the 5 × FAD group compared with the WT group; ^b^*p* < 0.05, ^bb^*p* < 0.001 the AAV-TaqTth-NT group compared with the WT group; ^c^*p* < 0.05 the AAV-TaqTth-T group compared with the 5 × FAD group; ^d^*p* < 0.05 the AAV-TaqTth-T group compared with the AAV-TaqTth-NT group in (**B**). **p < 0.01, ***p < 0.001 in (**D** and **E**). *ns* means no significant difference. **F**,** G** Effect of AAV-TaqTth-T on cognitive deficits in 5 × FAD mice assayed by NOR. The schematic shows the process of NOR (**F**) and NOR was assayed using the discrimination index (**G**). *n* = 8 mice. Data were analyzed by one-way ANOVA with Fisher’s LSD post hoc tests. mean ± s.e.m. **p < 0.01, ***p < 0.001. *ns* means no significant difference. **H** Immunoblotting shows the protein levels of HA-tagged TaqTth, Aβ, PSD-95, and synaptophysin in the hippocampus from 5 × FAD mice from 6 mice. GAPDH as a loading control.** I** Image shows IHC staining for EGFP (green) in AAV-infected cells in the hippocampus of 5 × FAD mice bilateral intrahippocampal injected with AAV-TaqTth-T. Nuclei (blue) were labeled by DAPI. **J**,** K** Representative images of the IHC for Aβ (red) in hippocampus from 5 × FAD mice. Nuclei (blue) were labeled by DAPI (**J**). Quantification of Aβ plaques number with different sizes (diameter < 20 μm, 20–40 μm and > 40 μm) per mm^2^ (**K**). *n* = 20 sections from 5 mice. Scar bar = 100 μm. Data were analyzed by one-way ANOVA with Fisher’s LSD post hoc tests. mean ± s.e.m. **p < 0.01, ***p < 0.001. *ns* means no significant difference. The data in the AAV-TaqTth-T, AAV-TaqTth-NT, and uninjection groups were shown by green square, brown circle, and pink triangle, respectively. **L**,** M** Representative images of the IHC for Iba1 (red) in hippocampus from 5 × FAD mice. Nuclei (blue) were labeled by DAPI (**L**). Quantification of the average number per mm^2^ with indicated sizes (diameter < 20 μm, 20–40 μm, and > 40 μm) of Iba1-positive microglial clusters (**M**). *n* = 20 sections from 5 mice. Scar bar = 100 μm. Data were analyzed by one-way ANOVA with Fisher’s LSD post hoc tests. mean ± s.e.m. ***p < 0.001. *ns* means no significant difference. The data in the AAV-TaqTth-T, AAV-TaqTth-NT, and uninjection groups were shown by green square, brown circle, and pink triangle, respectively. **N**,** O** Representative images of the IHC for GFAP (red) in hippocampus from 5 × FAD mice. Nuclei (blue) were labeled by DAPI (**N**). Quantification of the average number per mm^2^ with indicated sizes (diameter < 20 μm, 20–40 μm, and > 40 μm) of GFAP-positive astrocytic clusters (**O**). *n* = 20 sections from 5 mice. Scar bar = 100 μm. Data were analyzed by one-way ANOVA with Fisher’s LSD post hoc tests. mean ± s.e.m. *p < 0.05, ***p < 0.001. *ns* means no significant difference. The data in the AAV-TaqTth-T, AAV-TaqTth-NT, and uninjection groups were shown by green square, brown circle, and pink triangle, respectively

To study the cognitive function and AD pathologies of the 5 × FAD mice, we designed an experimental procedure as shown in a schematic (Fig. 5A). After injection of viral particles, the body and brain weight of mice was measured weekly, and no obvious change was found among the AAV-TaqTth-T-injected, AAV-TaqTth-NT-injected, and uninjected mice (Additional file 1: Fig. S11). 12 weeks after injection, the morris water maze (MWM) and novel object recognition (NOR) tests were conducted to assess the function of spatial learning and memory. As expected, in contrast to the WT, the AAV-TaqTth-NT-injected and uninjected 5 × FAD spent a longer time to find the hidden platform in the escape latency during 5 days of acquisition trials and the average number of platform crossing and time spent in the target quadrant were both reduced in probe trials (Fig. 5B to E). It is important to note that the 5 × FAD injected by AAV-TaqTth-T exhibited less time in the escape latency on the fourth and fifth day of acquisition trials compared to the AAV-TaqTth-NT-injected and uninjected mice (Fig. 5B), as well as increased number of platform crossing (Fig. 5C and E) and time spent in the target quadrant in probe trials (Fig. 5D). In the NOR tests, the WT spent more time on exploring a novel object than a previously recognized object, but the uninjected 5 × FAD spent nearly same time on exploring both objects (Fig. 5F, G), indicating a cognitive deficit in the 5 × FAD. Like the behavioral performance in MWM, the AAV-TaqTth-NT injection did not modify the behavioral performance of 5 × FAD mice in the NOR test. In contrast, the AAV-TaqTth-T injection largely improved the cognitive function in identifying novel objects (Fig. 5F, G). Together, these results demonstrate that the knockdown of mutated APP^swe^ by the AAV-TaqTth-T can adequately improve the cognitive function of the AD mice, suggesting a potential clinical application of AAV-TaqTth-T in AD treatment.

After 13 weeks of AAV-TaqTth-T and AAV-TaqTth-NT injection, mice were sacrificed for pathological study. Fluorescence images of EGFP showed a high transduction efficiency of AAV particles (Fig. 5I). Immunoblotting also indicated that the Aβ levels were decreased in the hippocampi (~ 52.7% compared to the uninjected mice, ~ 49.7% compared to the AAV-TaqTth-NT-injected mice) (Fig. 5H). We further analyzed the Aβ plaques with each different size (diameter: < 20 μm, 20–40 μm and > 40 μm) and found that the numbers of plaques with these three types of sizes were all considerably lowered in the hippocampi of the 5 × FAD mice injected with AAV-TaqTth-T (Fig. 5J, K). As known, Aβ plaques in the AD brains are surrounded by activated microglial cells and reactive astrocytes called microgliosis and astrogliosis, respectively. To evaluate the effect of AAV-TaqTth-T on gliosis, we examined the levels of microgliosis and astrogliosis, and immunohistochemical (IHC) analysis showed that the number of Iba1-positive (Iba1^+^, microglial marker) clusters in all sizes were reduced in AAV-TaqTth-T-injected 5 × FAD mice (Fig. 5L, M). Like microgliosis, AAV-TaqTth-T injection also significantly decreased the number of GFAP-positive (GFAP^+^, astrocyte marker) clusters in all sizes (Fig. 5N, O). The loss of synapses, as another Aβ-associated pathology, leads to cognitive decline in AD. Thereby, we detected the protein levels of synaptic markers including PSD-95 (a postsynaptic marker) and synaptophysin (a presynaptic marker). Immunoblotting showed that AAV-TaqTth-T injection, instead of AAV-TaqTth-NT injection, strongly augmented the levels of PSD-95 in the hippocampi, while mildly increased the expression of synaptophysin (Fig. 5H).

Taken together, our data infer that the AAV-TaqTth-hpRNA targeting *APP*^*swe*^ mRNA favorably reduces the neuropathology in AD mice.

### The compact TaqTth provides enough packaging capacity for APOE2 overexpression in a single AAV vector

**Fig. 6:**
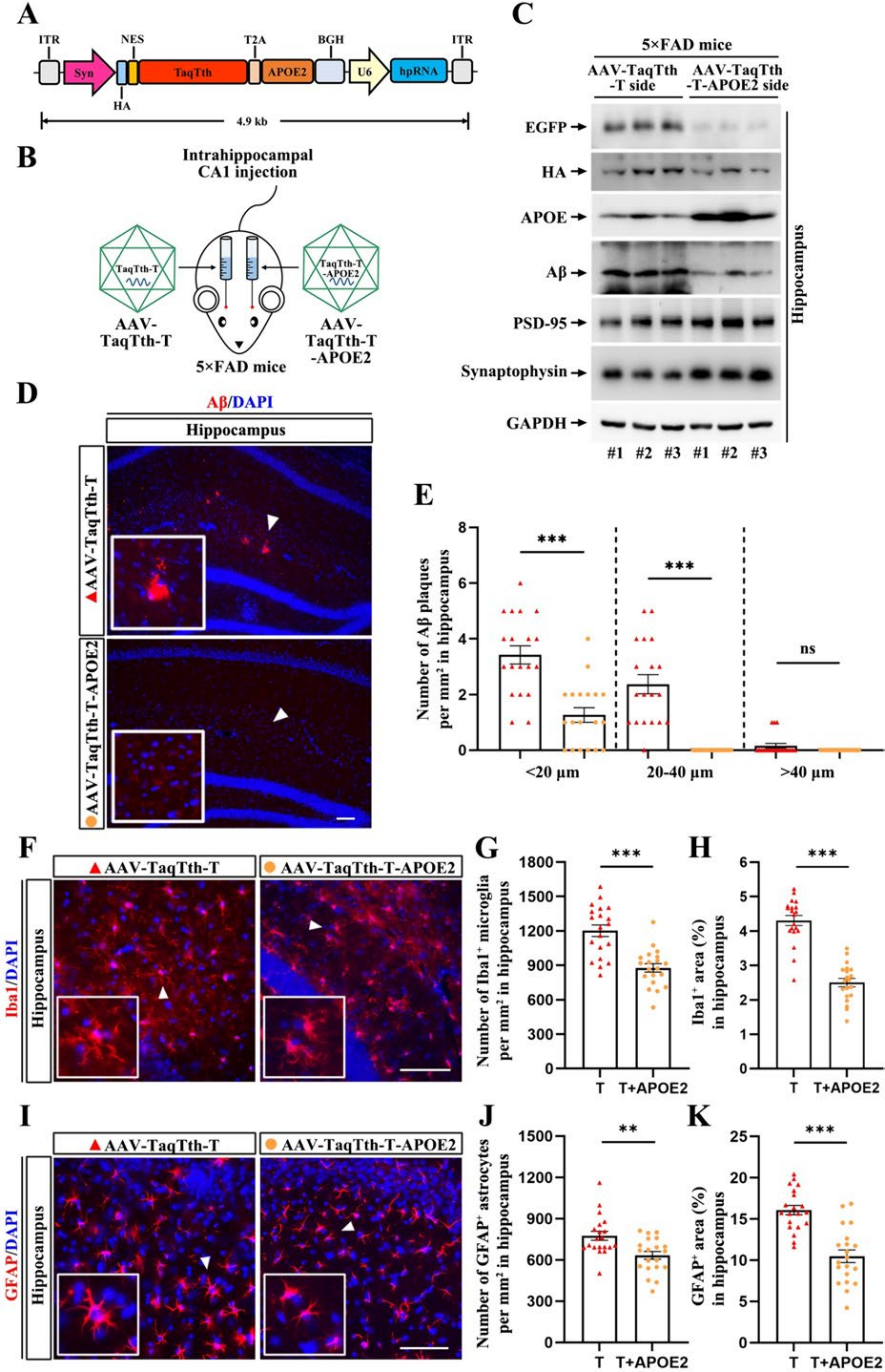
TaqTth combined with APOE2 overexpression eliminates Aβ pathologies in 5 × FAD mice. **A** A schematic of AAV-TaqTth-T-APOE2 vector shuttle plasmid. **B** Schematic shows intrahippocampal injection of AAV-TaqTth-T and AAV-TaqTth-T-APOE2 particles. **C** Immunoblotting shows the protein levels of EGFP (represent for AAV-TaqTth-T extendibility to the opposite side), HA (represent for the TaqTth expression), APOE, Aβ, PSD-95, and synaptophysin in the hippocampus from 3 mice. GAPDH as a loading control. **D**,** E** Representative images of the IHC for Aβ (red) and nuclei (blue) on different sides of the hippocampus with indicated virus injection. Scar bar = 100 μm. (**D**) Quantification of Aβ plaques number with different sizes (**E**) (diameter < 20 μm, 20–40 μm and > 40 μm) per mm^2^. Data represent mean ± s.e.m. ***p < 0.001. *ns* means no significant difference. The data in the AAV-TaqTth-T and AAV-TaqTth-T-APOE2 groups were shown by the red triangle and yellow circle, respectively. **F**,** G**,** H** Representative images of the IHC for Iba1 (red) (**F**) in the hippocampus of 5 × FAD mice with indicated virus injection. Nuclei (blue) were labeled by DAPI. Scar bar = 100 μm. Quantification of the average number per mm^2^ with Iba1-positive microglia (**G**) as well as the percentage of Iba1-positive (**H**) area. T: AAV-TaqTth-T, T + APOE2: AAV-TaqTth-T-APOE2. *n* = 20 sections from four mice. Data represent mean ± s.e.m. ***p < 0.001. The data in the AAV-TaqTth-T and AAV-TaqTth-T-APOE2 groups were shown by the red triangle and yellow circle, respectively. **I**,** J**,** K** Representative images of the IHC for GFAP (red) (**I**) in the hippocampus of 5 × FAD mice with indicated virus injection. Nuclei (blue) were labeled by DAPI. Scar bar = 100 μm. Quantification of the average number per mm^2^ with GFAP-positive astrocytes (**J**) as well as the percentage of GFAP-positive (**K**) area. T: AAV-TaqTth-T, T + APOE2: AAV-TaqTth-T-APOE2. *n* = 20 sections from four mice. Data represent mean ± s.e.m. **p < 0.01, ***p < 0.001. The data in the AAV-TaqTth-T and AAV-TaqTth-T-APOE2 groups were shown by the red triangle and yellow circle, respectively

Considering the much smaller size of TaqTth than Cas9, we calculated the free packaging capacity (the AAV vector genomes is about 4 kb after removing EGFP) and aimed to construct another AAV vector for possible better inhibition of AD pathology. Emerging evidence has suggested that AAV-mediated expression of APOE2 protects against AD pathogenesis in both preclinical and clinical studies [18, 19]. To this end, we cloned the APOE2 gene at downstream of HA-tagged TaqTth and fused with a T2A sequence, which allows translation skipping between these two proteins (Fig. 6A). We then used this vector to generate AAV9 particles (named as AAV-TaqTth-T-APOE2), following with one side intrahippocampal injection in 3-month-old 5 × FAD mice, and another opposite side injection of previously generated AAV-TaqTth-T as a control (Fig. 6B). As was expected, immunoblotting showed an elevated expression of APOE2 in the hippocampi injected with AAV-TaqTth-T-APOE2 (Fig. 6C). Thus, our TaqTth tool indeed offers enough free packaging space in AAV for APOE2 overexpression, likely will provide better efficacy for AD inhibition.

### AAV-TaqTth-T-APOE2 exerts a stronger reduction of AD pathologies than AAV-TaqTth-T

To compare the long-term efficacy of AAV-TaqTth-T-APOE2 and AAV-TaqTth-T, we injected AAV in AD mice for 3 months and then assessed the Aβ pathologies. Strikingly, the Aβ plaques with all different sizes were almost completely eliminated by AAV-TaqTth-T-APOE2 injection (Fig. 6D, E). In contrast to AAV-TaqTth-T, AAV-TaqTth-T-APOE2 further reduced the levels of Aβ by ~ 53.4% (Fig. 6C). In line with these data, the gliosis was also largely cleared by AAV-TaqTth-T-APOE2 injection as evidenced by a further decrease in the number of Iba1^+^ (Fig. 6F, G) and GFAP^+^ cells (Fig. 6I, J), as well as the area of the Iba1^+^ (Fig. 6F and H) and GFAP^+^ cells (Fig. 6I and K). In addition, the protein levels of PSD-95 and synaptophysin were also further increased by AAV-TaqTth-T-APOE2 compared to AAV-TaqTth-T itself (Fig. 6C). Simultaneously, we also compared the therapeutic effects of administering AAV-APOE2 alone with those of AAV-TaqTth-T-APOE2. It was found that in age-matched 5 × FAD mice, AAV-TaqTth-T-APOE2 exhibited significantly enhanced inhibitory effects compared to AAV-APOE2, including reductions in Aβ levels (Additional file 1: Fig. S12C) and plaque loads (Additional file 1: Fig. S12A, B), as well as the number of Iba1^+^ (Additional file 1: Fig. S12D, E) and GFAP^+^ (Additional file 1: Fig. S12G, H) cells and the area covered by the Iba1^+^ (Additional file 1: Fig. S12D and F) and GFAP^+^ (Additional file 1: Fig. S12G and I) cells. Correspondingly, the protein levels of PSD-95 and synaptophysin were remarkably higher in the AAV-TaqTth-T-APOE2 group than in the AAV-APOE2 group (Additional file 1: Fig. S12C).

Overall, these results suggest that the combination of the TaqTth and APOE2 overexpression packaged in a single AAV vector exhibits a better efficacy than TaqTth alone on the inhibition of AD pathologies.

## Discussion

**Fig. 7:**
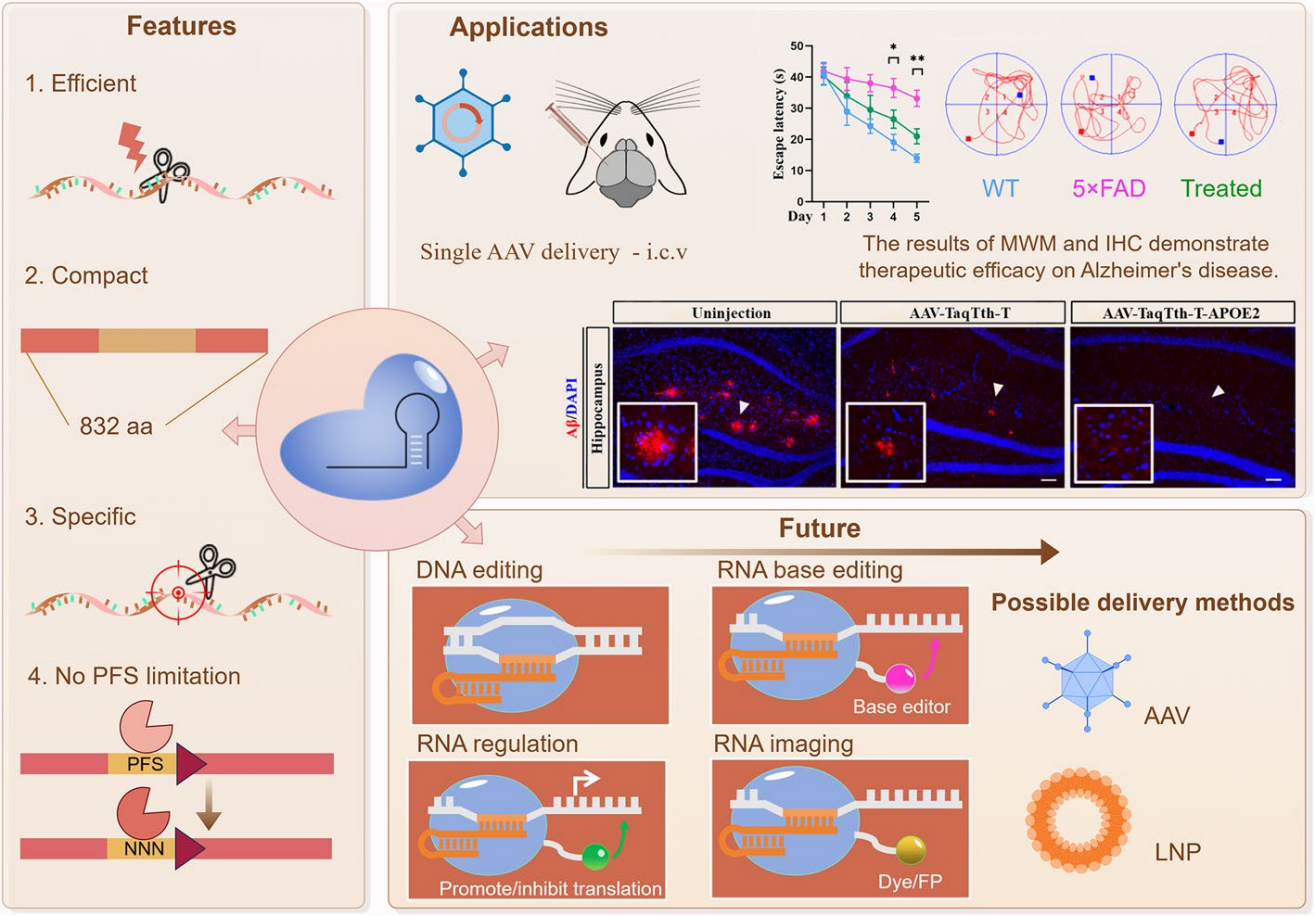
The summary of the findings and future directions of the TaqTth-hpRNA

In this study, a novel RNA cleaving system TaqTth-hpRNA, composed of compact TaqTth and hpRNAs, was developed and investigated here (Fig. 7). In vitro biochemical studies showed that the TaqTth-hpRNA could efficiently cleave artificially synthesized ssRNA. For mammalian transcripts, the TaqTth-hpRNA system cleaved with ~ 60% efficiency with the advantages of minimal flanking sequence-motif requirement and less cell viability damage. The transcriptome-wide RNA-seqs and mismatch tests reflected good specificity of TaqTth-hpRNA, which then was applied to mutant *APP*^*swe*^ mRNA silencing without altering the wild-type *APP* in AD. It was delivered by AAV vector in 5 × FAD mice and showed strong inhibition of pathologies. Notably, the combination with APOE2 overexpression encoded in a single AAV vector is available due to the compact size of TaqTth, and showed stronger inhibition of pathologies by silencing one “toxic” gene and expressing a second “protective” gene simultaneously. We also offer some guidelines to design hpRNA: (i) A 20–30-nt guide sequence is suggested. An appropriate Tm via benefits the binding of the probe with the target. (ii) The stem-loop part of the hpRNA can be fixed as designed here. (iii) Mutant bases in pathogenic mRNA could be placed at the 3’ terminal of the guide sequence in the hpRNA to avoid off-targeting the wild mRNA.

However, compared with the robust CRISPR-associated systems and siRNA, at present, the TaqTth-hpRNA is just a potential choice in some cases. In the past few years, Cas7 protein [20–22] from type III systems and Cas13 protein [23–25] from type VI systems have been harnessed for programmable RNA editing. The remaining concerns include collateral activity [26], the size of the nuclease, and the requirement of PFS. It is worth noting that several advanced CRISPR proteins have recently been developed, such as Cas13d [27] without PFS requirement, Cas13bt [28] and CasRx [29, 30] with smaller size, and Cas7-11 [31], Cas13d-N2V8 [16], or Cas13X-M17YY [16] with minimal collateral activity. Although the TaqTth-hpRNA has a small size and minimal flanking sequence-motif requirement, more work is still needed to further optimize the efficiency of RNA editing by testing more protein variants and hpRNA variants.

Previously, the OL-1, an antisense oligonucleotide (ASO) for APP, has shown improved cognitive function, lowered Aβ plaques, and reduced neuroinflammation [32]. An efficient siRNA design method [33] has been reported to improve the specificity, and short hairpin RNA (shRNA) targeting *APP* in APP/PS1 mice has been identified and shown benefits by reducing the deposition of Aβ plaques [34]. This reported APP allele-specific silencing [34] was achieved due to the “bulky” mismatched bases with favorable thermodynamic properties in the central placement of the shRNA used. However, as the author pointed out, it will not be the case for all dominantly acting alleles carrying nucleotide variations. The Cas9 system has been applied to the knock-out of *APP*^*swe*^ [35], which aims at the DNA level. The RfxCas13d system has also been used in silencing targeted mRNA to improve outcomes in the nervous system [36] (*SOD1* and *HTT* in a model of amyotrophic lateral sclerosis and Huntington’s disease, respectively). Owing to the challenge associated with viral vectors [17], recombinant AAV is currently the preferred viral vector for gene therapy due to its non-replicative nature and relatively low immunogenicity [37]. A new stereotype AAV-PHP.eB11 that crosses the BBB by intravenous injection has been developed [38]. Combined with the Cas9-mediated gene editing system, AAV-PHP.eB11 has shown advantages in AAV delivery into the brains [35]. Thus, we are also planning to utilize the AAV-PHP.eB11 vector in our future study to confirm the effect of the TaqTth-hpRNA on more pathogenic mRNA and hopefully the intravenous delivery of TaqTth-hpRNA will yield favorable benefits to clinical use.

The successful and efficient cleavage described in this study also encouraged us to investigate whether TaqTth-hpRNA can also effectively cleave bacterial RNA. Furthermore, the feature about whether TaqTth-hpRNA could be used for DNA editing is also worth exploring. The compact TaqTth and dTaqTth also provide an alternative to engineering efficient effectors with broad genome engineering applications, including RNA regulation, RNA base editing, and RNA imaging (Fig. 7).

## Conclusions

In summary, this research studied a new programmable RNA-editing tool, with the advantages of high activity, small size, minimal flanking sequence-motif requirement, and less cell viability damage. It was applied to mutant *APP*^*swe*^ mRNA silencing without altering the wild-type *APP* mRNA in Alzheimer’s disease to show strong inhibition of pathologies while showing the power of silencing one “toxic” gene and expressing a second “protective” gene by a single AAV vector. The results reported in this study that TaqTth-hpRNA has the potential as a useful alternative tool for the knockdown of pathogenic mRNA in gene therapy.

## Methods

### Cell lines

The cell lines were characterized by FuHeng Cell Center (Shanghai, China) using short tandem repeat (STR) markers.

### Construction of plasmid expressing TaqTth

Different constructs expressing TaqTth were generated. TaqTth ORF was synthesized and inserted into the pcDNA3.1( +), and the HA tag and NES were added to the N-terminus to generate pcDNA3.1( +)-HA-NES-TaqTth. For the *E. coli* sequence preference detection, TaqTth ORF was subcloned to a vector containing the Tc-inducible promoter, chloramphenicol-selectable marker, and p15A replication origin. For purified TaqTth in gel-experiments, TaqTth ORF was subcloned to the pET28a( +) to generate pET28a( +)-HA-NES-TaqTth.

### Designing and construction of the hairpin probes

The probe consisted of a stem-loop and a guide sequence. The guide sequence is reverse complementarity to its targeted loci sequence. The stem-loop sequence was fixed. The targeted loci sequences are shown in Additional file 2: Table S2. For RNA hairpin probes, a pU6-target was constructed. The backbone plasmid was digested by *Bbs* I and *Sac* I. Two partly reverse complementary oligos were synthesized and annealed by touch-down process (5’-CACCG NNNNNNNNNNNNNNNNNNNN aaa gtc ggc cga aag gcc gac ttt ttt AGCT -3’ and 5’- aaa aaa gtc ggc ctt tcg gcc gac ttt NNNNNNNNNNNNNNNNNNNN C-3’, N is the base in the guide sequence).

### Expression and purification of TaqTth recombinant protein

The pET28a( +)-HA-NES-TaqTth was transfected into host bacteria BL21(DE3), with the CaCl_2_-heat-shock method. Briefly, cells were cultured at 37 °C and induced with IPTG (0.1 mM) at 25 °C for 16 h to express TaqTth. The induced cells were collected, lysed with ultrasound, and centrifuged. TaqTth was purified from the crude extract by Ni-affinity chromatography for the His-tag in pET28a( +).

### Cleaving ssRNA in vitro

A 10-μL reaction mixture consisting of hpRNA (10 pmol) (Additional file 2: Table S1), single-strand RNA substrate (ssRNA) (10 pmol) (Additional file 2: Table S1), Tris–HCl (pH 8.9) 100 mM, KCl 500 mM, MgCl_2_ (7.5 mM), and an appropriate amount of enzyme was prepared and incubated at 37 °C for 2 h. The 5’ end of the target ssRNA was labeled with fluorescent FAM. The tips, tubes, and reagents used in this experiment were all treated with DEPC (diethyl pyrocarbonate).

### Denatured-polyacrylamide gel electrophoresis (PAGE)

The products derived from the above reactions were analyzed with PAGE under denaturing conditions. The loading buffer contained 95% formamide, 0.025% SDS, 0.025% bromophenol blue, 0.025% xylene cyanol FF, 0.025% ethidium bromide, and 0.5 mM EDTA. The samples were then loaded onto a 20% PAGE gel at room temperature and ran in a buffer containing urea (8.7 M) and Tris-borate (89 mM). The electrophoresis was run at 9.6 V/cm for 2 h. The gel was imaged by Amersham Imager 600 (GE Healthcare).

### Structure simulation of protein and probe

The amino acid sequence of TaqTth was carried out on SWISS-MODEL website (swissmodel.expasy.org, GMQE = 0.91). The Ramachandran plots and sequence coverage images show good confidence. Use Alphafold Server to simulate protein-probe binding (Alphafoldserver.com). The elements include the sequence of TaqTth, hpRNA, targeted RNA, and two Mg^2+^ (RNA sequences do not affect docking).

### Electrophoretic mobility shift assay (EMSA)

A 10-μL reaction mixture consisting of hpRNA (10 pmol), MOPS (10 mM), 0.05% Tween-20, 0.01% nonidet P-40, MgCl_2_ (7.5 mM), and the suitable amount of TaqTth/dTaqTth was incubated at 37 °C for 0.5 h. The 5’ end of the hpRNA was labeled with fluorescent group Cy5. The reacting products were analyzed with 12% native-PAGE. After electrophoresis, the gel was imaged by Amersham Imager 600 (GE Healthcare).

### Sequence preference detection

An oligo sequence, with two degeneracy bases at the 5' end, a target in the middle, and four degeneracy bases at the 3' end, was designed and inserted into the ampicillin start codon to construct a plasmid library. The ampicillin-resistant plasmid library, the chloramphenicol-resistant TaqTth protein expression plasmid and the spectinomycin-resistant probe plasmid were transferred into the electrical natural competence by bio-red electroporation. The expression of TaqTth was induced by the Tet inducer. Then 16 h later, cells were scraped off plates and extracted plasmid DNA. The unrelated probe NT was used as the control group. To assess sequence preference, the nucleotide portion of the original library containing the target sequence was extracted and PCR-specific amplification was performed. The specific PCR product was sequenced by Sangon Biotech (Shanghai, China).

### TaqTth-hpRNA assay

Cells were maintained in Dulbecco’s modified Eagle’s medium (DMEM) supplemented with 10% fetal bovine serum (HyClone), 100 U/mL penicillin, and 100 µg/mL streptomycin at 37 °C with 5% CO_2_ incubation. For 24-well plates, 80,000 cells per well were plated. Plasmids of pcDNA3.1( +)-HA-NES-TaqTth (800 ng per well) and pU6-target (1000 ng per well) were transfected by TransIntro™ EL Tranfection Reagent following the manufacturer’s recommended protocol. After 48 or 72 h, cells were harvested to perform the following experiments.

### siRNA assay

The siRNAs were synthesized by Sangon Biotech company. For 24-well plates, 80,000 cells per well were plated. The 50 pmol of siRNA were transfected per well. After 48 or 72 h, cells were harvested to perform the following experiments.

### CRISPR assay

For 24-well plates, 80,000 cells per well were plated. Plasmids of CRISPR-LwCas13a-msfGFP-NES (800 ng per well) (pC0056, Addgene plasmid #105815) and LwCas13a guide expression backbone with U6 promoter (1000 ng per well) (pC016, Addgene plasmid #91906) were transfected by TransIntro™ EL Tranfection Reagent following the manufacturer’s recommended protocol. After 48 or 72 h, cells were harvested to perform the following experiments.

### Flow cytometry

Cells were resuspended in PBS, and the fluorescence intensity EGFP (488 nm excitation and 525 nm emission) or mCherry (587 nm excitation and 610 nm emission) was measured immediately using FACSCalibur (Becton Dickinson). The data was analyzed by FlowJo software.

### Quantitative PCR

Cells were trypsinized and washed once with RNA-easy Isolation Reagent (Vazyme, R701-01/02) following the manufacturer’s instructions. The cDNA synthesis was performed using the HiScript III 1st Strand cDNA Synthesis Kit (+ gDNA wiper) (Vazyme, R312-01). The cDNA was used in quantitative PCR analyses with AceQ qPCR SYBR Green Master Mix (Low ROX Premixed) (Vazyme, Q131-02) with specific primers listed in Additional file 2: Table S3.

### RNA sequencing

Total RNA was extracted using the Total RNA Extractor (Trizol) kit (B511311, Sangon, China) according to the manufacturer’s protocol and treated with RNase-free DNase I to remove genomic DNA contamination. RNA integrity was evaluated with a 1.0% agarose gel. Thereafter, the quality and quantity of RNA were assessed using a NanoPhotometer® spectrophotometer (IMPLEN, CA, USA) and a Qubit® 2.0 Flurometer (Invitrogen). The high-quality RNA samples were subsequently submitted to the Sangon Biotech (Shanghai) Co., Ltd. for library preparation and sequencing. A total amount of 1 μg RNA per sample was used as input material for the RNA sample preparations. Sequencing libraries were generated using VAHTS™ mRNA-seq V2 Library Prep Kit for Illumina® following manufacturer’s recommendations and index codes were added to attribute sequences to each sample. Briefly, mRNA was purified from total RNA using poly-T oligo-attached magnetic beads. Fragmentation was carried out using divalent cations under elevated temperature in VAHTS™ First Strand Synthesis Reaction Buffer. First-strand cDNA was synthesized using random hexamer primer and M-MuLV Reverse Transcriptase (RNase H-). Second-strand cDNA synthesis was subsequently performed using DNA polymerase I and RNase H. Remaining overhangs was converted into blunt ends via exonuclease/polymerase activities. After the adenylation of 3’ ends of DNA fragments, the adaptor was ligated to prepare for the library. To select cDNA fragments of preferentially 150 ~ 200 bp in length, the library fragments were purified with AMPure XP system (Beckman Coulter, Beverly, USA). Then 3-μL USER Enzyme (NEB, USA) was used with size-selected, adaptor-ligated cDNA at 37 °C for 15 min followed by 5 min at 95 °C before PCR. Then PCR was performed with Phusion High-Fidelity DNA polymerase, universal PCR primers, and Index (X) Primer. At last, PCR products were purified (AMPure XP system) and library quality was assessed on the Agilent Bioanalyzer 2100 system. The libraries were then quantified and pooled. Paired-end sequencing of the library was performed on the NovaSeq sequencers (Illumina, San Diego, CA) Gene expression values of the transcripts were computed by StringTie (version 1.3.3b). Principal component analysis (PCA) and principal co-ordinates analysis (PCoA) were performed to reflect the distance and difference between samples. The TPM (transcripts per million) eliminates the influence of gene lengths and sequencing discrepancies to enable direct comparison of gene expression between samples.

### CCK8 and cell proliferation assay

On day 1, cells were seeded into 96-well plates (Corning) at a density of 6000 cells per well. On day 2, cells were transfected with siRNA (10 pmol) / the plasmid of TaqTth (120 ng) plus the plasmid of hpRNA (80 ng)/the plasmid of Cas13a (120 ng) plus the plasmid of gRNA (80 ng), respectively. Then 48 h after transfection, 10-μL CCK8 solution was added to each well and incubated for 2 h at 37 °C. Absorbance was read at 450 nm with a Synergy™ HT multi-mode reader (Bio-Tek, Winoosky, VT). Cell morphology was observed under a tenfold microscope before and after transfection. The views selected randomly were photographed and calculated the proportion of cell area using Image J.

### Immunofluorescence (IF)

Cells were fixed with 4% paraformaldehyde in PBS at 1-h intervals, permeabilized in 0.2% (v/v) TritonX-100 for 20 min, and blocked with 3% BSA for 30 min. Incubation with primary antibodies against HA tag (Abclonal Technology, #AE008, 1:200) was done overnight at 4 °C. The coverslips were washed three times with PBS and followed by co-incubation with goat anti-mouse IgG (H + L) cross-adsorbed secondary antibody, Alexa Fluor™ 488 (ThermoFisher, #A11001) for 1 h at 37 °C. The nuclei were stained with 4’,6-diamidino-2-phenylindole (DAPI) for 20 min before imaging. The coverslips were inverted onto slides and immersed in a mounting medium. A laser scanning confocal microscope FV10-ASW [Ver 2.1] (Olympus Corp, MPE FV1000) was used for co-localization analysis.

### Western blot (WB)

For cell tests, protein samples were isolated with lysis buffer, eluted with SDS buffer, separated by SDS–polyacrylamide gels, and electroblotted onto PVDF membranes. The specific protein bands were stained with High-sig ECL Western Blotting Substrate (Tanon) and imaged using the Amersham Imager 600 (GE Healthcare). Primary antibodies included antibodies against HA (abclonal, #AE006, 1:2000) and GAPDH (abclonal, #AC033, 1:5000).

For animal tests, the protein samples of hippocampus were extracted using a kit (Genuin Biotech, #415) in accordance with the kit instructions after mice were sacrificed and perfused with saline. The primary antibodies included antibodies against HA (cell signaling technology, #3724, 1:2000), APP (proteintech, #25524–1-AP, 1:1000), Aβ (cell signaling technology, #2454, 1:2000), PSD-95 (cell signaling technology, #3450, 1:2000), synaptophysin (cell signaling technology, #36406, 1:2000), APOE (Academy Bio-Medical, #50H-G1a, 1:1000), EGFP (Santa Cruze, #sc-9996, 1:2000), and GAPDH (abclonal, #AC033, 1:2000).

### Preparation of adeno-associated virus (AAV)

The original vector pAAV CMV-ZsGreen U6-gRNA (Addgene, #163021) was reconstructed for our experiments. Briefly, the CMV promoter and ZsGreen were replaced by CAG or Syn promoter and EGFP respectively. Then, the T2A linked HA-NES-TaqTth was added following EGFP. The targeting hpRNA or non-targeting hpRNA were cloned after U6 promoter. For AAV-TaqTth-T-APOE2 vector, the EGFP was deleted to reduce the size of the vector. For AAV-APOE2 vector, the T2A linked APOE2 was added following EGFP. AAV is packaged and purified in accordance with the Addgene website’s instructions (https://www.addgene.org/protocols/aav-production-hek293-cells/; https://www.addgene.org/protocols/aav-purification-iodixanol-gradient-ultracentrifugation/).

### Mice and intrahippocampal injection

5 × FAD (JAX, Stock # 034848) mice co-express mutant forms of human APP (the Swedish mutation: K670N/M671L; the Florida mutation: I716V; the London mutation: V717I) and PS1 (M146L; L286V) and C57BL/6 mice were used in this study. In some cases, 3-month-old 5 × FAD mice were randomly divided into three groups, bilateral intrahippocampal injected with or without AAV-TaqTth-T or AAV-TaqTth-NT. In some cases, 3-month-old 5 × FAD mice were randomly divided into two groups, bilateral intrahippocampal injected with AAV-APOE2 or AAV-TaqTth-T-APOE2. In other cases, 3-month-old 5 × FAD mice were intrahippocampal injected of AAV-TaqTth-T on one side and injected of AAV-TaqTth-NT or AAV-TaqTth-T-APOE2 on the opposite side. For intrahippocampal injection, 2-μL viral particles (10^12^ ~ 10^13^ v.g./mL) were injected into the hippocampal CA1 region at -2.2 mm from bregma, mediolateral ± 1.7 mm, depth 2.4 mm of mouse brains. All mice were maintained in a temperature (26-28 °C)- and humidity-controlled environment with a 12-h light–dark cycle and free access to food and water unless otherwise indicated. All experiments were conducted in accordance with the regulations for the Administration of Affairs Concerning Experimental Animals and approved by the Laboratory Animal Care Committee at the China Pharmaceutical University (permit number SYXK-2021-0011).

### Morris water maze (MWM) and novel object recognition (NOR)

Mice were habituated to the procedure room 24 h before each behavioral test. The Morris water maze (MWM) test was performed as described [39]. Briefly, in a circular pool (130 cm in diameter, 60-cm deep), filled to a depth of 30 cm, and maintained at a temperature of roughly 22 °C, mice were trained to locate a hidden escape platform that was 10 cm in diameter and 1 cm below the water’s surface. The southwest quadrant’s center was designated as the location of the escape platform. The training lasted for 5 days and involved four trail mice per day. At various entry points (north, east, south-east, and north-west), the mice were released towards the pool wall in a different order every day. Swimming was automatically video-tracked for up to 60 s or until the subject reached the concealed escape platform and stayed there for at least 2 s. Mice that took longer than 60 s to locate the escape platform were led there until they spent at least 10 s there. After acquisition trials, the escape platform was removed before mice were released facing to the pool wall at the north-east entry point. In probe trials, swimming was video-tracked for 60 s automatically.

Novel object recognition (NOR) test was performed. Briefly, mice were firstly placed in a 45 cm × 45 cm × 40 cm open field box for 2 min of habituation. Post 30 min of rest following habituation, two identical (same color set) objects were placed in the area in a fixed location for the mice to explore for 8 min as training. After 60 min of rest following training, one object was replaced with a different color, and the mice were allowed to explore them in the box for an additional 8 min as test trials. Alcohol was used to remove odors from the floor and the objects during habituation, training, and test trials. The time spent perusing each object was recorded automatically by a digital camera. Calculate the discrimination index, using the equation: Discrimination index = *T*_Novel_** /** (*T*_Novel_ + *T*_Familiar_).

### Immunohistochemistry (IHC)

In preparation for cryostat sectioning, mice were sacrificed and brains were isolated, fixed in 4% PFA, and dehydrated. Leica CM1950 microtome was used to cut cryosections at 30 μm. After that, IHC was performed according to our standard protocol as described previously [40]. The primary antibodies included antibodies against Aβ (cell signaling technology, #2454, 1:500), Iba1 (GeneTex, #019-19741, 1:500), and GFAP (abcam, #ab7260, 1:500). The second antibodies included anti-goat Alexa546-labeled (Invitrogen, #A10040, 1:500).

### Statistical analysis

All data were expressed as mean ± s.d. or mean ± s.e.m. from independent experiments performed in a parallel manner. Statistical tests included one-tailed or two-tailed unpaired two-sample *t*-test, one-way, or two-way ANOVA. *p* < 0.05 were considered statistically significant.

### Supplementary Information

Additional file 1: Fig. S1. TaqTth and hpRNA binding validation. Fig. S2. The sequence of the U6-promoting hpRNA probe in the pU6-target plasmid. Fig. S3. The sequence of the recombinant TaqTth protein coding sequence. Fig. S4. The expression of recombinant HA-NES-TaqTth or HA-TaqTth-NES in mammalian cells. Fig. S5. The EGFP fluorescence knockdown in cells heterologously expressing the NES-TaqTth or TaqTth-NES. Fig. S6. The EGFP fluorescence knockdown in EGFP-stable cells transfected with a single plasmid encoding the TaqTth and hpRNA with a mcherry tag. Fig. S7. The dTaqTth and TaqTth-mediated knockdown of KRAS. Fig. S8. The analysis of the total RNA treated by the TaqTth-hpRNA, Cas13a, and siRNA strategies by agarose electrophoresis. Fig. S9. Single mismatch assay for specificity analysis. Fig. S10. A single AAV9 vector shuttle plasmid and validation. Fig. S11. The body and brain weight of AAV-TaqTth-T-injected, AAV-TaqTth-NT-injected and uninjected 5×FAD mice. Fig. S12. APOE2 overexpression and combinaition with TaqTth eliminate Aβ pathologies in 5×FAD mice.

Additional file 2: Table S1. The targeted ssRNA and hpRNAs in gel experiments. Table S2. The targeted mRNA loci for assays in cells and in vivo. Table S3. The primers for qPCR. Table S4. The targeted mRNA loci for gRNA and siRNA.

Additional file 3. Uncropped images of Western blots.

Additional file 4. Review history.

## Data Availability

All data generated or analyzed during this study are included in this article and its Supplementary Information files. The sequencing data from this study have been deposited in the Gene Expression Omnibus (GEO) repository, with the accession numbers GSE269592 and GSE269593 [41, 42]. The flow cytometry data from this study have been deposited on the web (http://flowrepository.org) [43–45]. The uncropped images of gels are shown in Additional file 3.
