# Supplementary material for: TaqTth-hpRNA: a novel compact RNA-targeting tool for specific silencing of pathogenic mRNA
Source: Genome Biol. 2024 Jul 7;25:179. doi: 10.1186/s13059-024-03326-3 (PMC11229350; doi:10.1186/s13059-024-03326-3)

**Fig. S1. TaqTth and hpRNA binding validation.** (A). The graph provided by AlphaFold Server shows the RNA probe and target sections in brown and the TaqTth protein sections in blue, Mg^2+^ in green. (B). The electrophoretic mobility shift assay (EMSA) of TaqTth and dTaqTth. Different migration velocities of Cy5-modified hpRNAs with purified TaqTth and dTaqTth protein.

**Fig. S2.** **The sequence of the U6-promoting hpRNA probe in the pU6-target plasmid.** The part of U6 promoter was marked in lower cases. The restriction sites *Bbs* I and *Sac* I used were labeled in italics. The part of guide-sequence was underlined. The part of the stem-loop was marked in red. Two T bases (lowercase with underlined) were added to assist in transcription termination.

gagggcctatttcccatgattccttcatatttgcatatacgatacaaggctgttagagagataattggaattaatttgactgtaaacacaaagatattagtacaaaatacgtgacgtagaaagtaataatttcttgggtagtttgcagttttaaaattatgttttaaaatggactatcatatgcttaccgtaacttgaaagtatttcgatttcttggctttatatatcttgtggaaaggac***GAAACACCG*NNNNNNNNNNNNNNNNNNNNAAAGTCGGCCGAAAGGCCGACTTTTtt*AGCTC***

**Fig. S3. The sequence of the recombinant TaqTth protein coding sequence.** The recombinant TaqTth protein (832 amino acids) is fused by two domains from *Thermus aquaticus* polymerase and one domain from *Thermus thermophilus* polymerase. The domain from *Thermus thermophilus* polymerase was underlined.

The nucleic acid sequence:

ATGAGGGGGATGCTGCCCCTCTTTGAGCCCAAGGGCCGGGTCCTCCTGGTGGACGGCCACCACCTGGCCTACCGCACCTTCCACGCCCTGAAGGGCCTCACCACCAGCCGGGGGGAGCCGGTGCAGGCGGTCTACGGCTTCGCCAAGAGCCTCCTCAAGGCCCTCAAGGAGGACGGGGACGCGGTGATCGTGGTCTTTGACGCCAAGGCCCCCTCCTTCCGCCACGAGGCCTACGGGGGGTACAAGGCGGGCCGGGCCCCCACGCCGGAGGACTTTCCCCGGCAACTCGCCCTCATCAAGGAGCTGGTGGACCTCCTGGGGCTGGCGCGCCTCGAGGTCCCGGGCTACGAGGCGGACGACGTCCTGGCCAGCCTGGCCAAGAAGGCGGAAAAGGAGGGCTACGAGGTCCGCATCCTCACCGCCGACAAAGACCTTTACCAGCTCCTTTCCGACCGCATCCACGCCCTCCACCCCGAGGGCTACCTCATCACCCCGGCCTGGCTTTGGGAAAAGTACGGCCTGAGGCCCGACCAGTGGGCCGACTACCGGGCCCTGACCGGGGACGAGTCCGACAACCTTCCCGGGGTCAAGGGCATCGGGGAGAAGACGGCGAGGAAGCTTCTGGAGGAGTGGGGGAGCCTGGAAGCCCTCCTCAAGAACCTGGACCGGCTGAAGCCCGCCATCCGGGAGAAGATCCTGGCCCACATGGACGATCTGAAGCTCTCCTGGGACCTGGCCAAGGTGCGCACCGACCTGCCCCTGGAGGTGGACTTCGCCAAAAGGCGGGAGCCCGACCGGGAGAGGCTTAGGGCCTTTCTGGAGAGGCTTGAGTTTGGCAGCCTCCTCCACGAGTTCGGCCTTCTGGAAAGCCCCAAGGCCCTGGAGGAGGCCCCCTGGCCCCCGCCGGAAGGGGCCTTCGTGGGCTTTGTGCTTTCCCGCAAGGAGCCCATGTGGGCCGATCTTCTGGCCCTGGCCGCCGCCAGGGACGGCCGGGTGCACCGGGCAGCAGACCCCTTGGCGGGGCTAAAGGACCTCAAGGAGGTCCGGGGCCTCCTCGCCAAGGACCTCGCCGTCTTGGCCTCGAGGGAGGGGCTAGACCTCGTGCCCGGGGACGACCCCATGCTCCTCGCCTACCTCCTGGACCCCTCCAACACCACCCCCGAGGGGGTGGCGCGGCGCTACGGGGGGGAGTGGACGGAGGACGCCGCCCACCGGGCCCTCCTCTCGGAGAGGCTCCATCGGAACCTCCTTAAGCGCCTCGAGGGGGAGGAGAAGCTCCTTTGGCTCTACCACGAGGTGGAAAAGCCCCTCTCCCGGGTCCTGGCCCACATGGAGGCCACCGGGGTACGGCGGGACGTGGCCTACCTTCAGGCCCTTTCCCTGGAGCTTGCGGAGGAGATCCGCCGCCTCGAGGAGGAGGTCTTCCGCTTGGCGGGCCACCCCTTCAACCTCAACTCCCGGGACCAGCTGGAAAGGGTGCTCTTTGACGAGCTTAGGCTTCCCGCCTTGGGGAAGACGCAAAAGACAGGCAAGCGCTCCACCAGCGCCGCGGTGCTGGAGGCCCTACGGGAGGCCCACCCCATCGTGGAGAAGATCCTCCAGCACCGGGAGCTCACCAAGCTCAAGAACACCTACGTGGACCCCCTCCCAAGCCTCGTCCACCCGAGGACGGGCCGCCTCCACACCCGCTTCAACCAGACGGCCACGGCCACGGGGAGGCTTAGTAGCTCCGACCCCAACCTGCAGAACATCCCCGTCCGCACCCCCTTGGGCCAGAGAATCCGCCGGGCCTTCATCGCCGAGGAGGGGTGGCTATTGGTGGCCCTGGACTATAGCCAGATAGAGCTCAGGGTGCTGGCCCACCTCTCCGGCGACGAGAACCTGATCCGGGTCTTCCAGGAGGGGCGGGACATCCACACGGAGACCGCCAGCTGGATGTTCGGCGTCCCCCGGGAGGCCGTGGACCCCCTGATGCGCCGGGCGGCCAAGACCATCAACTTCGGGGTCCTCTACGGCATGTCGGCCCACCGCCTCTCCCAGGAGCTAGCCATCCCTTACGAGGAGGCCCAGGCCTTCATTGAGCGCTACTTTCAGAGCTTCCCCAAGGTGCGGGCCTGGATTGAGAAGACCCTGGAGGAGGGCAGGAGGCGGGGGTACGTGGAGACCCTCTTCGGCCGCCGCCGCTACGTGCCAGACCTAGAGGCCCGGGTGAAGAGCGTGCGGGAGGCGGCCGAGCGCATGGCCTTCAACATGCCCGTCCAGGGCACCGCCGCCGACCTCATGAAGCTGGCTATGGTGAAGCTCTTCCCCAGGCTGGAGGAAATGGGGGCCAGGATGCTCCTTCAGGTCCACGACGAGCTGGTCCTCGAGGCCCCAAAAGAGAGGGCGGAGGCCGTGGCCCGGCTGGCCAAGGAGGTCATGGAGGGGGTGTATCCCCTGGCCGTGCCCCTGGAGGTGGAGGTGGGGATAGGGGAGGACTGGCTCTCCGCCAAGGAGTGA

The amino acid sequence:

MRGMLPLFEPKGRVLLVDGHHLAYRTFHALKGLTTSRGEPVQAVYGFAKSLLKALKEDGDAVIVVFDAKAPSFRHEAYGGYKAGRAPTPEDFPRQLALIKELVDLLGLARLEVPGYEADDVLASLAKKAEKEGYEVRILTADKDLYQLLSDRIHALHPEGYLITPAWLWEKYGLRPDQWADYRALTGDESDNLPGVKGIGEKTARKLLEEWGSLEALLKNLDRLKPAIREKILAHMDDLKLSWDLAKVRTDLPLEVDFAKRREPDRERLRAFLERLEFGSLLHEFGLLESPKALEEAPWPPPEGAFVGFVLSRKEPMWADLLALAAARDGRVHRAADPLAGLKDLKEVRGLLAKDLAVLASREGLDLVPGDDPMLLAYLLDPSNTTPEGVARRYGGEWTEDAAHRALLSERLHRNLLKRLEGEEKLLWLYHEVEKPLSRVLAHMEATGVRRDVAYLQALSLELAEEIRRLEEEVFRLAGHPFNLNSRDQLERVLFDELRLPALGKTQKTGKRSTSAAVLEALREAHPIVEKILQHRELTKLKNTYVDPLPSLVHPRTGRLHTRFNQTATATGRLSSSDPNLQNIPVRTPLGQRIRRAFIAEEGWLLVALDYSQIELRVLAHLSGDENLIRVFQEGRDIHTETASWMFGVPREAVDPLMRRAAKTINFGVLYGMSAHRLSQELAIPYEEAQAFIERYFQSFPKVRAWIEKTLEEGRRRGYVETLFGRRRYVPDLEARVKSVREAAERMAFNMPVQGTAADLMKLAMVKLFPRLEEMGARMLLQVHDELVLEAPKERAEAVARLAKEVMEGVYPLAVPLEVEVGIGEDWLSAKE-

**Fig. S4.** **The expression of recombinant HA-NES-TaqTth or HA-TaqTth-NES in mammalian cells.** *Negative control* means the group transfected with only empty vector.

**Fig. S5. The EGFP fluorescence knockdown in cells heterologously expressing the NES-TaqTth or TaqTth-NES.** The schematic diagram process of the EGFP assay (A), the relative EGFP fluorescence (B) and the percentages of EGFP-positive cells (C) in groups treated by the NES-TaqTth or TaqTth-NES. ** p < 0.01. *ns* means no significant difference. *NT* means the non-targeting.

**Fig. S6. The EGFP fluorescence knockdown in EGFP-stable cells transfected with a single plasmid encoding the TaqTth and hpRNA with a mcherry tag.** ** p < 0.01. *** p < 0.001. *NT* means the non-targeting.

**Fig. S7. The dTaqTth and TaqTth-mediated knockdown of KRAS.** mean ± s.e.m. n=3. * p < 0.05. *ns* means no significant difference. *NT* means the non-targeting.

**Fig. S8.** **The analysis of the total RNA treated by the TaqTth-hpRNA, Cas13a, and siRNA strategies by agarose electrophoresis.**

**Fig. S9. Single mismatch assay for specificity analysis.** A panel of hpRNAs (Hp-EGFP) with a single-nucleotide mutation is shown below. Editing efficiencies are quantified as the geometric mean of EGFP fluorescence intensity. mean ± s.e.m. n=3. Mismatches are shown in red. ****p < 0.0001.

**
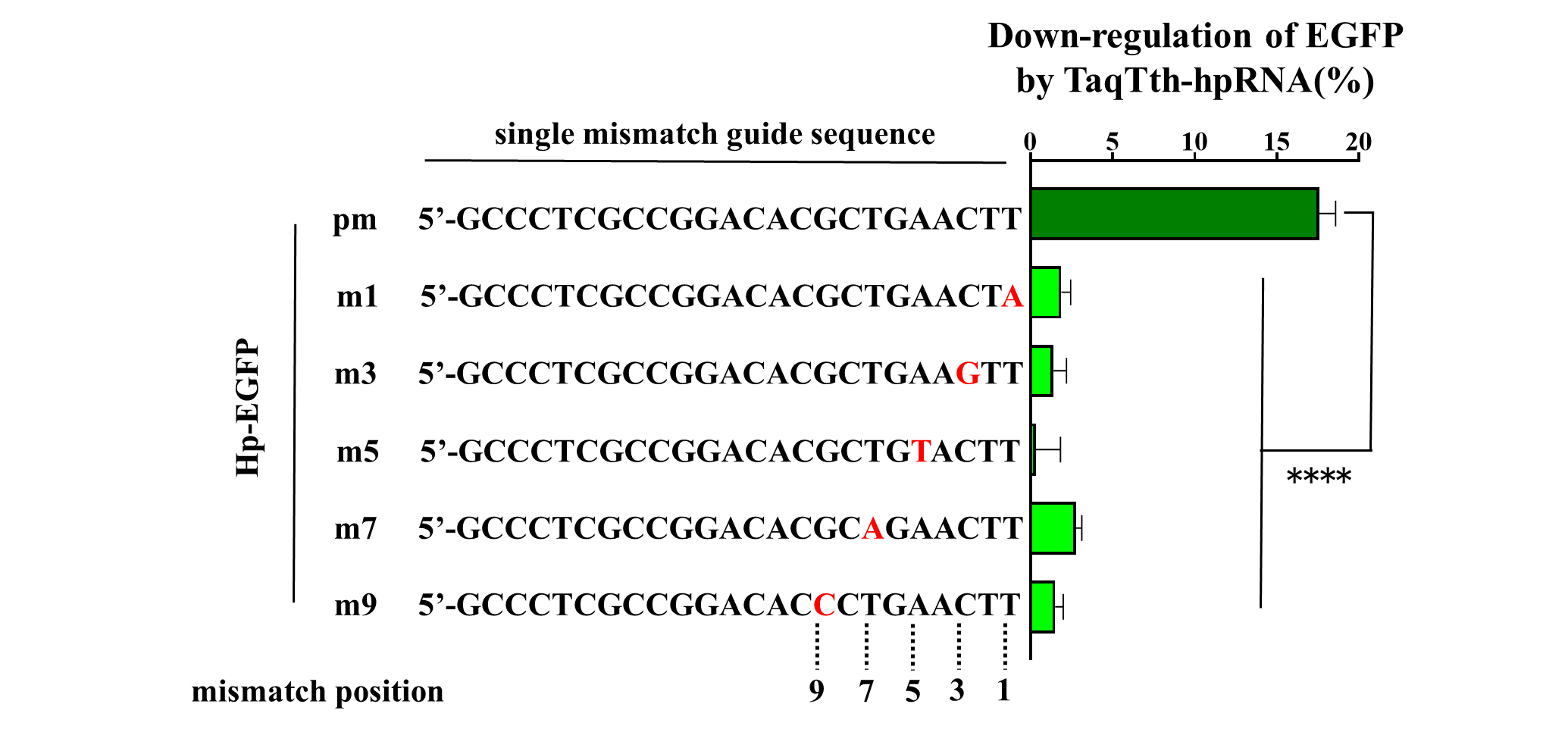
**

**Fig. S10.** **A single AAV9 vector shuttle plasmid and validation.** The schematic (A). The EGFP-TaqTth expression in HEK293T cells (B). The knockdown efficiencies of shuttle plasmids in the HEK293T cells over-expressing *APP^wt^* or *APP^swe^* in mRNA level. (C) and protein level (D). mean ± s.e.m. n=3. ***p* < 0.01. *ns* means no significant difference. *NT* means the non-targeting.

**Fig. S11. The body (A) and brain (B) weight of AAV-TaqTth-T-injected, AAV-TaqTth-NT-injected and uninjected 5×FAD mice.** *ns* means no significant difference. *NT* means the non-targeting.

**Fig. S12. APOE2 overexpression and combinition with TaqTth eliminate Aβ pathologies in 5×FAD mice.** Representative images of the IHC for Aβ (red) and nuclei (blue) in hippocampus from different groups of mice with indicated virus injection (A). Quantification of Aβ plaques number with different sizes (B) (diameter<20 μm, 20-40 μm and >40 μm) per mm^2^. n = 20 sections from 3 mice. Scar bar = 100 μm. Immunoblotting shows the protein levels of Aβ, PSD-95 and Synaptophysin in hippocampus from AAV-APOE2-injected and AAV-TaqTth-T-APOE2-injected groups (C). n = 3 mice per group. GAPDH as a loading control. Representative images of the IHC for Iba1 (red) (D) in hippocampus of 5×FAD mice with indicated virus injection. Nuclei (blue) were labeled by DAPI. Scar bar = 100 μm. Quantification of the average number per mm^2^ with Iba1-positive microglia (E) as well as the percentage of Iba1-positive (F) area. APOE2: AAV-APOE2, T+APOE2: AAV-TaqTth-T-APOE2. n = 20 sections from 3 mice. Representative images of the IHC for GFAP (red) (G) in hippocampus of 5×FAD mice with indicated virus injection. Nuclei (blue) were labeled by DAPI. Scar bar = 100 μm. Quantification of the average number per mm^2^ with GFAP-positive astrocytes (H) as well as the percentage of GFAP-positive (I) area. APOE2: AAV-APOE2, T+APOE2: AAV-TaqTth-T-APOE2. n = 20 sections from 3 mice. Scar bar = 100 μm. Data represent mean ± s.e.m. ****p* < 0.001. The data in the AAV-APOE2 and AAV-TaqTth-T-APOE2 groups were shown by dark red triangle and yellow circle, respectively.


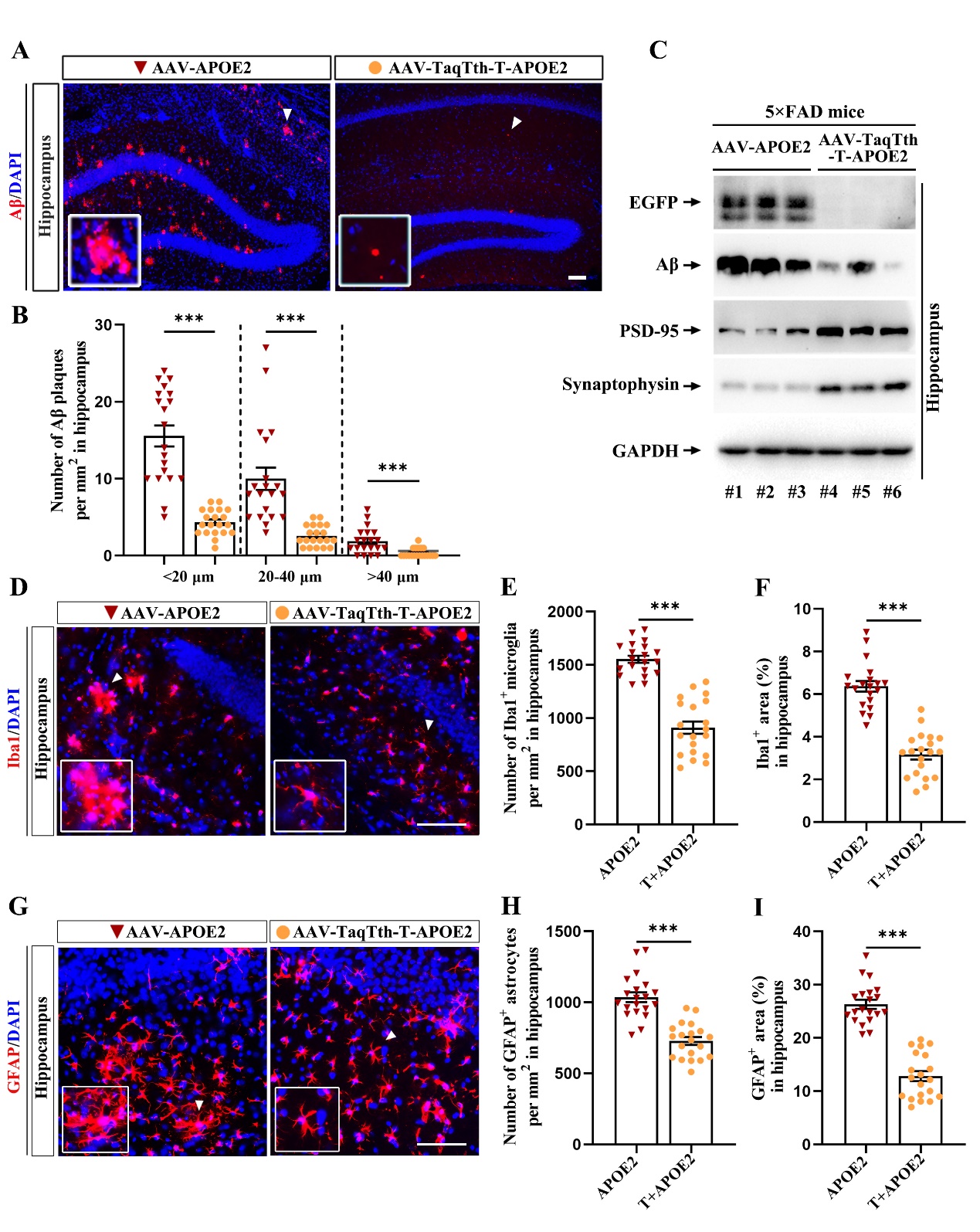

Supplement: Supplementary file 1 — Additional file 1: Fig. S1. TaqTth and hpRNA binding validation. Fig. S2. The sequence of the U6-promoting hpRNA probe in the pU6-target plasmid. Fig. S3. The sequence of the recombinant TaqTth protein coding sequence. Fig. S4. The expression of recombinant HA-NES-TaqTth or HA-TaqTth-NES in mammalian cells. Fig. S5. The EGFP fluorescence knockdown in cells heterologously expressing the NES-TaqTth or TaqTth-NES. Fig. S6. The EGFP fluorescence knockdown in EGFP-stable cells transfected with a single plasmid encoding the TaqTth and hpRNA with a mcherry tag. Fig. S7. The dTaqTth and TaqTth-mediated knockdown of KRAS. Fig. S8. The analysis of the total RNA treated by the TaqTth-hpRNA, Cas13a, and siRNA strategies by agarose electrophoresis. Fig. S9. Single mismatch assay for specificity analysis. Fig. S10. A single AAV9 vector shuttle plasmid and validation. Fig. S11. The body and brain weight of AAV-TaqTth-T-injected, AAV-TaqTth-NT-injected and uninjected 5×FAD mice. Fig. S12. APOE2 overexpression and combinaition with TaqTth eliminate Aβ pathologies in 5×FAD mice. [file 13059_2024_3326_MOESM1_ESM.docx]
