# Supplementary material for: TaqTth-hpRNA: a novel compact RNA-targeting tool for specific silencing of pathogenic mRNA
Source: Genome Biol. 2024 Jul 7;25:179. doi: 10.1186/s13059-024-03326-3 (PMC11229350; doi:10.1186/s13059-024-03326-3)
Supplement: Supplementary file 2 — Additional file 2: Table S1. The targeted ssRNA and hpRNAs in gel experiments. Table S2. The targeted mRNA loci for assays in cells and in vivo. Table S3. The primers for qPCR. Table S4. The targeted mRNA loci for gRNA and siRNA. [file 13059_2024_3326_MOESM2_ESM.docx]

**Table S1. The artificially synthesized targeted ssRNA and hpRNAs in gel experiments.** The part of the stem-loop was marked in lowercase letters.

| **Locus** | **5’-3’ sequence** |
| --- | --- |
| ssRNA target | FAM-CAGCAGUUUGGCCCGCCCAAAAUCUGUGAUCUUGACAUG  or  CAGCAGUUUGGCCCGCCCAAAAUCUGUGAUCUUGACAUG-FAM |
| hp-st1 | AAGAUCACAGAUUUUGGGCGagagucggccuuuuggccgacucuc |
| hp-st2 | AAGAUCACAGAUUUUGGGCGaaagucggccgaaaggccgacuuuu |
| hp-10 nt | AUUUUGGGCGaaagucggccgaaaggccgacuuuu |
| hp-15 nt | CACAGAUUUUGGGCGaaagucggccgaaaggccgacuuuu |
| hp-20 nt | Same as hp-st2 |
| hp-25 nt | AUGUCAAGAUCACAGAUUUUGGGCGaaagucggccgaaaggccgacuuuu |
| hp-cp-1 | CAUGUCAAGAUCACAGAUUUaaagucggccgaaaggccgacuuuu |
| hp-cp-2 | Same as hp-st2 |
| hp-cp-3 | AUCACAGAUUUUGGGCGGGCaaagucggccgaaaggccgacuuuu |

**Table S2. The targeted mRNA loci for assays in cells *and in vivo*.**

| **Locus** | **5’-3’ sequence** |
| --- | --- |
| egfp mRNA T1 | GGUCGAGCUGGACGGCGACGUAAACGGC |
| egfp mRNA T2 | CCACCCUGACCUACGGCGUGCAGUGCUU |
| egfp mRNA T3 | CUUCAAGGACGACGGCAACUACAAGACC |
| egfp mRNA T4 | UAUCAUGGCCGACAAGCAGAAGAACGGC |
| egfp mRNA T5 | CCCAACGAGAAGCGCGAUCACAUGGUCC |
| kras mRNA T1 | AUGACUGAAUAUAAACUUGU |
| kras mRNA T2 | UCCUACAGGAAGCAAGUAGU |
| kras mRNA T3 | AAAUCAUUUGAAGAUAUUCACCAUUAUA |
| kras mRNA T4 | GAAAUAAAUGUGAUUUGCCU |
| kras mRNA T5 | GAGGAUGCUUUUUAUACAUU |
| kras mRNA T6 | AAAAAUGCAUUAUAAUGUAA |
| ppib mRNA T1 | AUGCUGCGCCUCUCCGAACG |
| ppib mRNA T2 | GCCCAAAGUCACCGUCAAGG |
| ppib mRNA T3 | ACAGCAAAUUCCAUCGUGUAAUCAAGGA |
| ppib mRNA T4 | GCGCUUCCCCGAUGAGAACUUCAAACUG |
| ppib mRNA T5 | UCUUCAUCACGACAGUCAAG |
| ppib mRNA T6 | UUGCCAUCGCCAAGGAGUAG |
| app mRNA T-12 | UCUGGAUGCAGA |
| app mRNA T-14 | UCUGGAUGCAGAAU |
| app mRNA T-16 | UCUGGAUGCAGAAUUC |
| app mRNA T-18 | UCUGGAUGCAGAAUUCCG |
| app mRNA T-20 | UCUGGAUGCAGAAUUCCGAC |

**Table S3. The primers for qPCR.**

| **gene** | **5’-3’ sequence** |
| --- | --- |
| KRAS | F: ACAGTAGACACAAAACAGGCTCAGG |
|  | R: CCTCCACTCTCTGTCTTGTCTTTGC |
| PPIB | F: AGGAGGAAAGAGCATCTACGGTGAG |
|  | R: CAGGCTGTCTTGACTGTCGTGATG |
| APP | F: GAAGTGGCTGAGGTGGAAGAAGAAG |
|  | R: AGACTCTGTGGTGGTGGTGGTG |
| GAPDH | F: TAGTGGAAGGACTCATGACC |
|  | R: TCCACCACCCTGTTGCTGTA |

**Table S4. The targeted mRNA loci for gRNA and siRNA.**

| **Locus** | **5’-3’ sequence** |
| --- | --- |
| gRNA-egfp-T1 | GCCGUUUACGUCGCCGUCCAGCUCGACC |
| gRNA-egfp-T2 | AAGCACUGCACGCCGUAGGUCAGGGUGG |
| gRNA-egfp-T3 | GGUCUUGUAGUUGCCGUCGUCCUUGAAG |
| gRNA-egfp-T4 | GCCGUUCUUCUGCUUGUCGGCCAUGAUA |
| gRNA-egfp-T5 | GGACCAUGUGAUCGCGCUUCUCGUUGGG |
| gRNA-kras | GAAAUAAAUGUGAUUUGCCU |
| siRNA-ppib-1 | GCGCUUCCCCGAUGAGAACUUCAAACUG |
| siRNA-ppib-2 | UCUUCAUCACGACAGUCAAG |
| siRNA-kras | GAAAUAAAUGUGAUUUGCCU |
| siRNA-app | UCUGGAUGCAGAAUUCCGAC |
