# Supplementary material for: TaqTth-hpRNA: a novel compact RNA-targeting tool for specific silencing of pathogenic mRNA
Source: Genome Biol. 2024 Jul 7;25:179. doi: 10.1186/s13059-024-03326-3 (PMC11229350; doi:10.1186/s13059-024-03326-3)
Supplement: Supplementary file 3 — Additional file 3. Uncropped images of Western blots. [file 13059_2024_3326_MOESM3_ESM.pdf]

Figure 1D

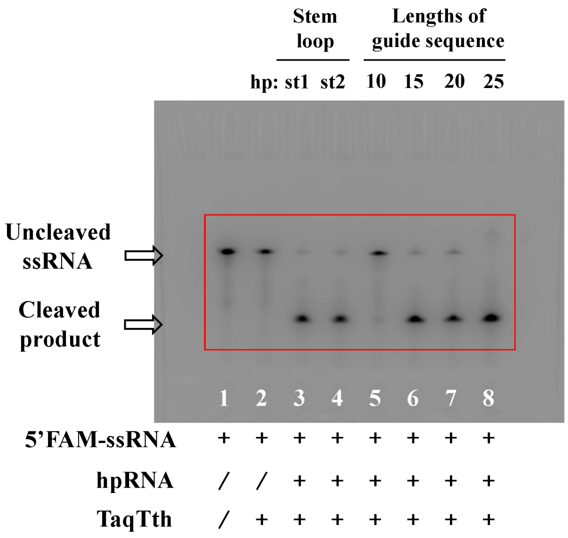
Stem loop
Lengths of guide sequence
hp: st1 st2 10 15 20 25
Uncleaved ssRNA
Cleaved product
1 2 3 4 5 6 7 8
5'FAM-ssRNA + + + + + + + +
hpRNA / / + + + + + +
TaqTth / + + + + + + +

Figure 1E

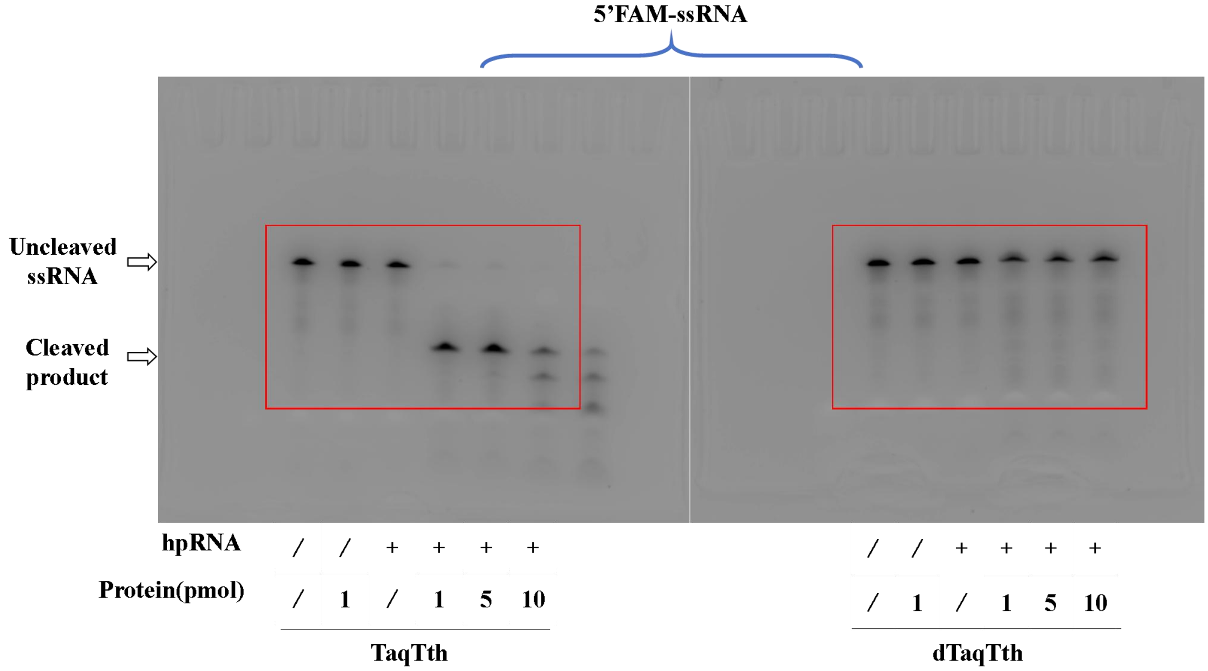
5'FAM-ssRNA
Uncleaved ssRNA
Cleaved product
hpRNA / / + + + +
Protein(pmol) / 1 / 1 5 10
TaqTth
hpRNA / / + + + +
Protein(pmol) / 1 / 1 5 10
dTaqTth

Figure 1F

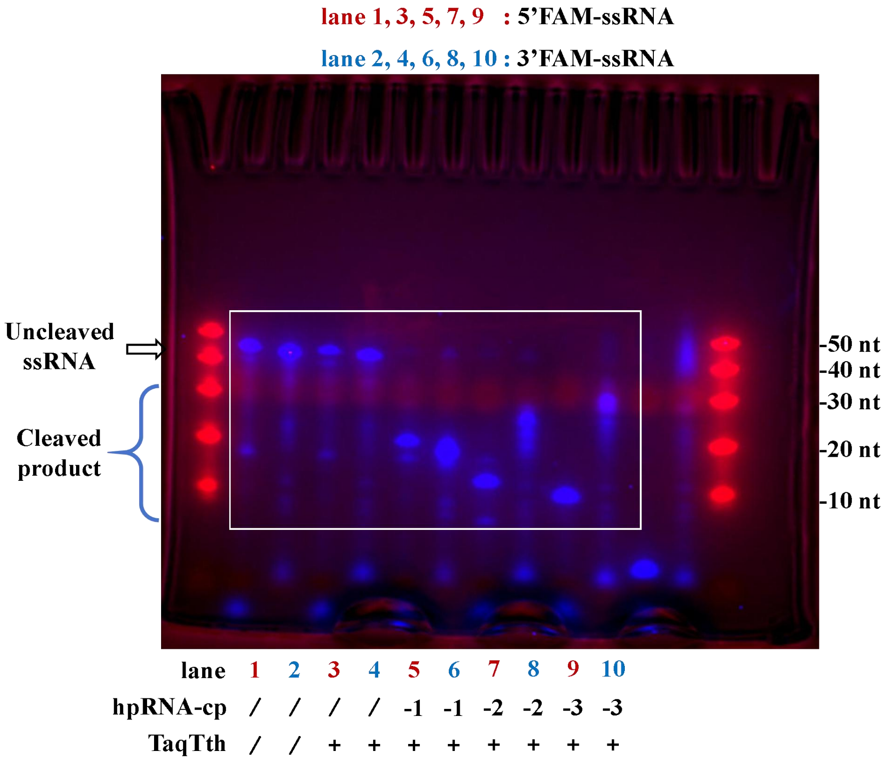
lane 1, 3, 5, 7, 9 : 5'FAM-ssRNA
lane 2, 4, 6, 8, 10 : 3'FAM-ssRNA
Uncleaved ssRNA
Cleaved product
-50 nt
-40 nt
-30 nt
-20 nt
-10 nt
lane 1 2 3 4 5 6 7 8 9 10
hpRNA-cp / / / / -1 -1 -2 -2 -3 -3
TaqTth / / + + + + + + + +

Figure 4E

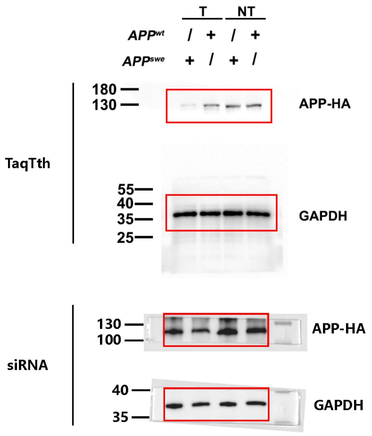
T
NT
APPwt / + / +
APPswe + / + /
180
130
APP-HA
TaqTth
55
40
35
25
GAPDH
130
100
APP-HA
siRNA
40
35
GAPDH

Figure 4F

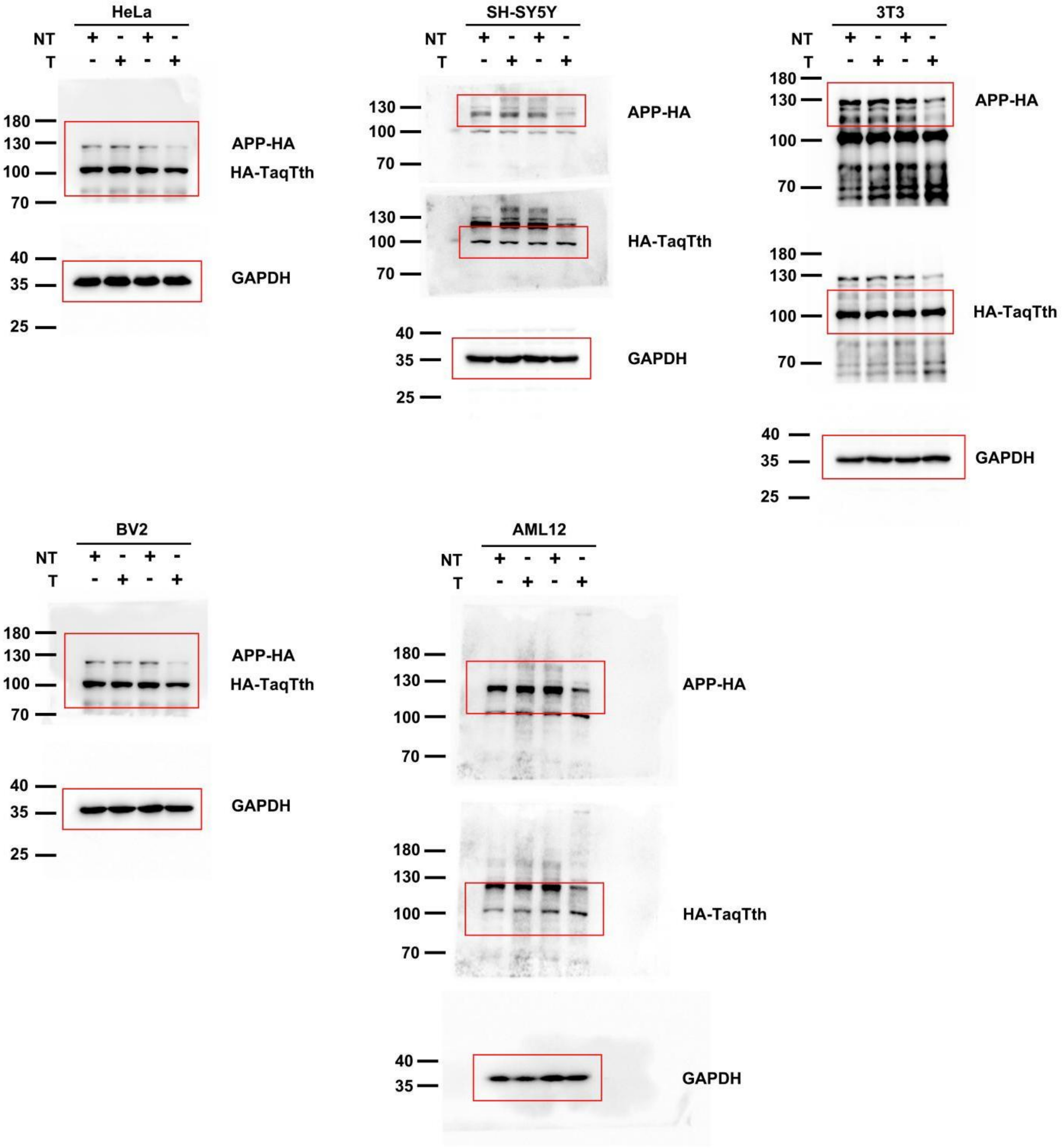
HeLa
NT + - + -
T - + - +
180
130
100
70
APP-HA
HA-TaqTth
40
35
25
GAPDH
SH-SY5Y
NT + - + -
T - + - +
130
100
70
APP-HA
130
100
70
HA-TaqTth
40
35
25
GAPDH
3T3
NT + - + -
T - + - +
180
130
100
70
APP-HA
180
130
100
70
HA-TaqTth
40
35
25
GAPDH
BV2
NT + - + -
T - + - +
180
130
100
70
APP-HA
HA-TaqTth
40
35
25
GAPDH
AML12
NT + - + -
T - + - +
180
130
100
70
APP-HA
180
130
100
70
HA-TaqTth
40
35
GAPDH

Figure 4I

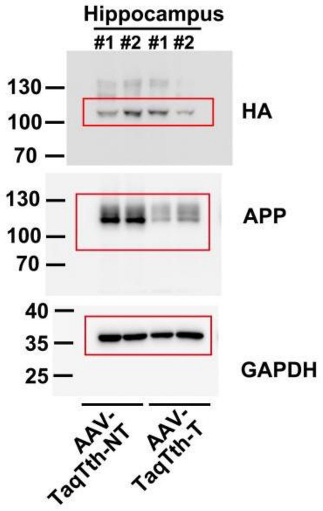

Hippocampus
#1 #2 #1 #2
130
100
70
HA
130
100
70
APP
40
35
25
GAPDH
AAV-TaqTth-NT
AAV-TaqTth-T

Figure 5H

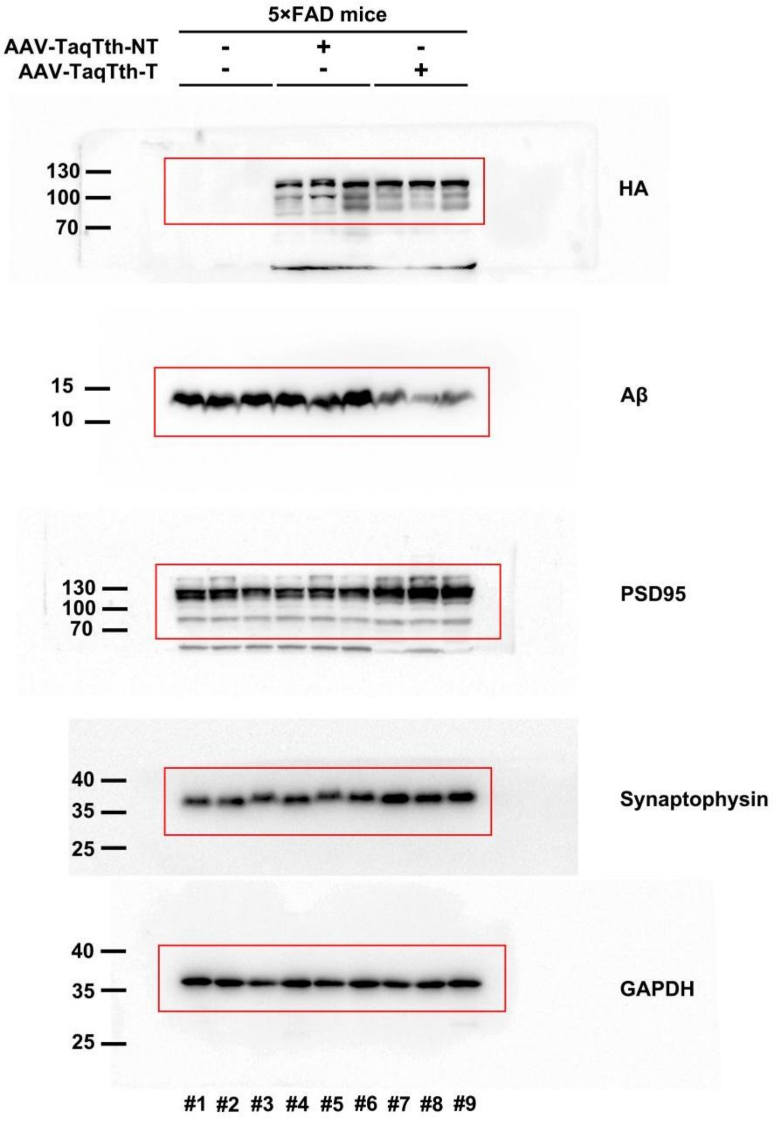

5×FAD mice
AAV-TaqTth-NT - + -
AAV-TaqTth-T - - +
130
100
70
HA
15
10
Aβ
130
100
70
PSD95
40
35
25
Synaptophysin
40
35
25
GAPDH
#1 #2 #3 #4 #5 #6 #7 #8 #9

Figure 6C

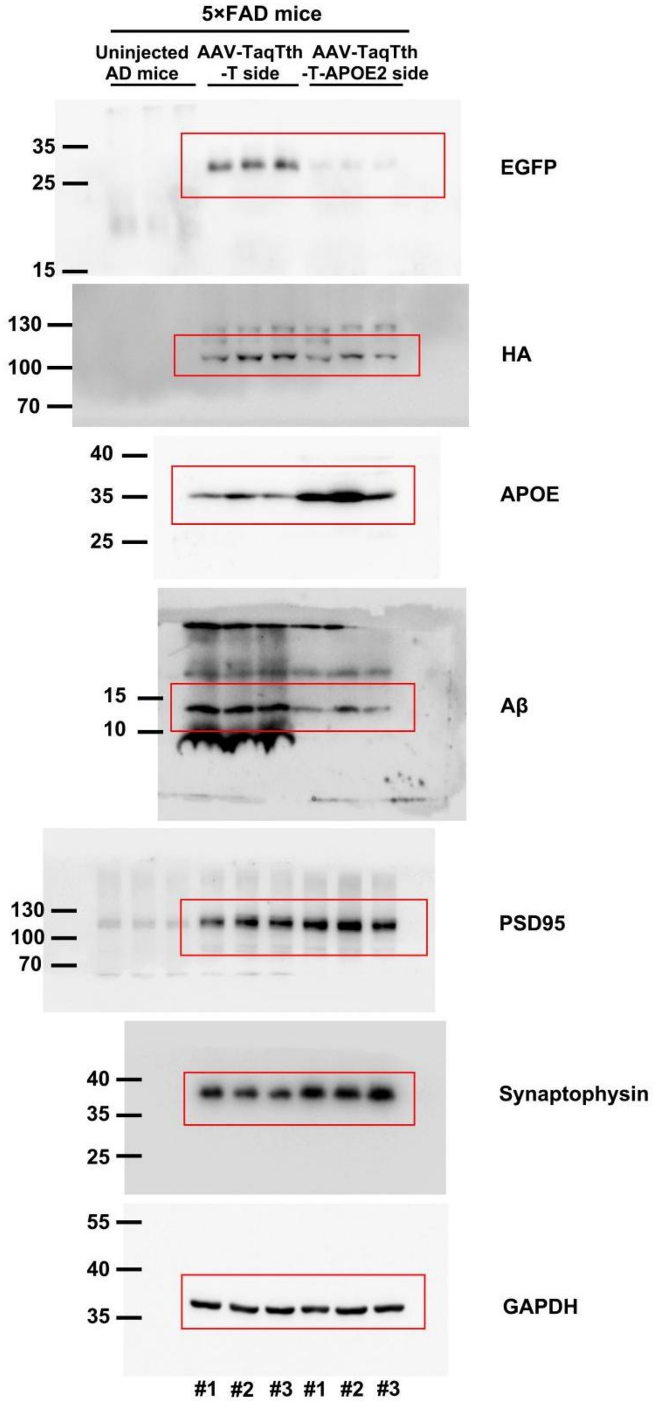
5×FAD mice
Uninjected AD mice
AAV-TaqTth -T side
AAV-TaqTth -T-APOE2 side
35
25
15
EGFP
130
100
70
HA
40
35
25
APOE
15
10
Aβ
130
100
70
PSD95
40
35
25
Synaptophysin
55
40
35
GAPDH
#1 #2 #3 #1 #2 #3

Figure S4

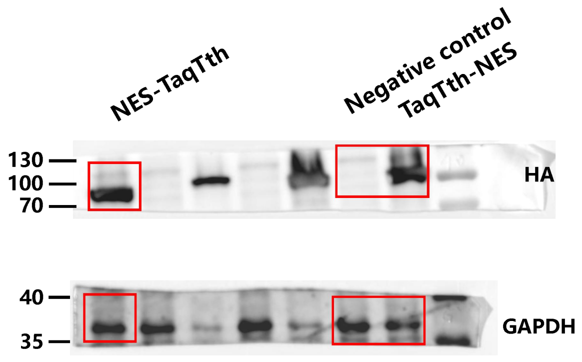

NES-TaqTth
Negative control
TaqTth-NES
130
100
70
HA
40
35
GAPDH

Figure S10D

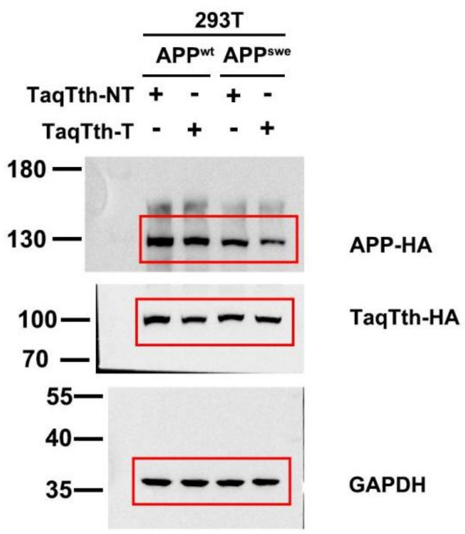

293T
APPwt
APPswe
TaqTth-NT + - + -
TaqTth-T - + - +
180
130
APP-HA
100
70
TaqTth-HA
55
40
35
GAPDH

Figure S12C

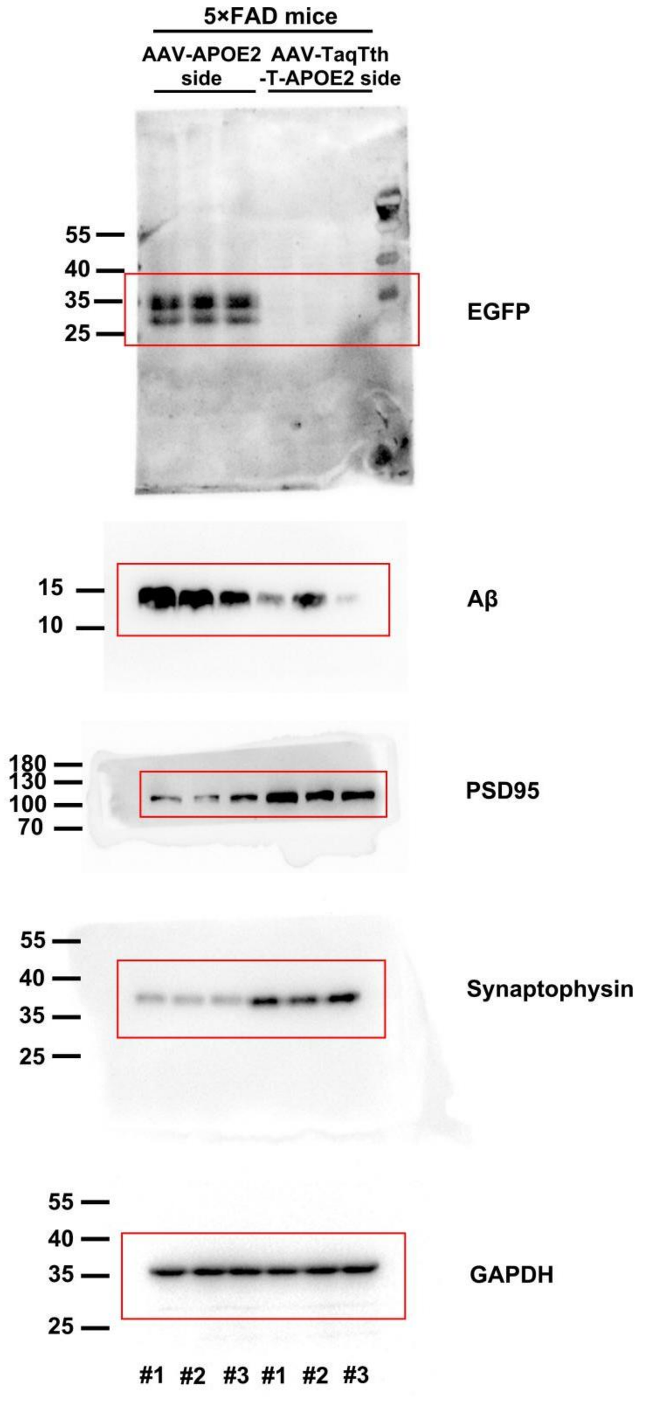
5×FAD mice
AAV-APOE2 side
AAV-TaqTth -T-APOE2 side
55
40
35
25
EGFP
15
10
Aβ
180
130
100
70
PSD95
55
40
35
25
Synaptophysin
55
40
35
25
GAPDH
#1 #2 #3 #1 #2 #3
