# Supplementary material for: TaqTth-hpRNA: a novel compact RNA-targeting tool for specific silencing of pathogenic mRNA
Source: Genome Biol. 2024 Jul 7;25:179. doi: 10.1186/s13059-024-03326-3 (PMC11229350; doi:10.1186/s13059-024-03326-3)
Supplement: Supplementary file 4 — Additional file 4. Review history. [file 13059_2024_3326_MOESM4_ESM.docx]

Review History

**First round of review**

**Reviewer 1**

**Were you able to assess all statistics in the manuscript, including the appropriateness of statistical tests used?**

No.

**Were you able to directly test the methods?**

No.

**Comments to author:**

In the manuscript, the authors developed and tested a novel RNA-interfering tool (TagTth-hpRNA) to knockdown targeted mRNA, which was used to cleave mutant APPswe mRNA without altering the wild-type APP mRNA in Alzheimer's disease. The developed RNA knockdown tool is very interesting. However, the characterization of this tool is insufficient, comparison between the TagTth-hpRNA and the CRISPR/Cas13 system is not described, some experiments are preliminary, and some critical controls are missing, all of which damages the conclusions.

Major concerns:

1. Figure 1 showed the idea of the design of the TagTth-hpRNA. Based on reference 5 (DOI: 10.1074/jbc.M002268200), the authors created the TagTth-hpRNA system. However, no in vitro characterization experiments were performed. Did the tool perform as expected?

a) What was the cutting site? What were the critical amino acid residues for the cutting? Mutation at these critical residues could create "dead" mutant, which was an essential control for core experiments, in addition to the NT control.

b) What was the design principle for the Stem-Loop on the hpRNA (the content or the length of the stem or the loop)? What is the basis for selecting the Stem-Loop version used in the manuscript? Did the length of the guide sequence affect RNA cleavage? What was the optimal length?

c) The authors did similar experiments in their previous articles that reported the development of other new editing tools (Nucleic Acids Res 2020, Genome Biol 2016), why such experiments were missing in the current work, even the TagTth-hpRNA tool were claimed better than the precious ones by the authors themselves?

2. Figure 2 showed "the feasibility of cleaving mRNA guided by TaqTth-hpRNA in mammalian cells". However, without any in vitro or in vivo "cleavage" assays, it was hard to claim RNA "cleavage" (cleavage dead mutant was a critical control to draw such a conclusion).

a) The EGFP reporter was transiently co-transfected with the TagTth-hpRNA system, and it is well known that many factors would affect the performance of transiently transfected reporter, an EGFP stable cell line or knock-in cell line would lead to more reliable data. And even on the stable reporter cell line, the plasmids encoding TaqTth and hpRNA should be traced by different colors of fluorescent proteins. FACS analyses should gated according to these colors. In addition, as for the FACS data in Figure 2B and 2C, the EGFP-negative and the EGFP-positive populations were not clearly displayed.

b) The target site for kras mRNA T6 was "5'-AAAAAUGCAUUAUAAUGUAA-3'", therefore, the DNA encoding hpRNA should be the complementary and reverse sequence "5'-TTACATTATAATGCATTTTT-3'". As we know, 5-6 Ts is the stop signal for U6 promoter-driven transcription, thus the Stem-Loop in the hpRNA could not be transcripted. However, KRAS T6 showed 56% knockdown efficiency. If this hold true, how did the TagTth-hpRNA work? This observation again required more detailed in vitro characterization of this system.

c) Similar to CRISPR system, the authors also tried to determine whether the TagTth-hpRNA system required a PAM sequence as shown in Figure 2G. However, the number of target sequence was too small to draw a conclusion. Generally, a library contains sufficient sequence should be edited by the TagTth-hpRNA system in vitro or in E.coli, and deep-seq was performed before and after editing, the depleted sequences were the edited ones and PAM could be discovered in these sequences. Please refer to the articles from Dr. Jennifer Doudna or Dr. Feng Zhang or other scientists.

3. Figure 3: since TagTth-hpRNA system was more similar to CRISPR/Cas13 than siRNA, the comparison between TagTth-hpRNA and Cas13 should be shown. In Figure 3D and 3F, the inhibition of PP1B was less than 20%, which mean that there was only very weak inhibition even on the target gene. Therefore, it is reasonable that no off-target effect was observed. A more effective target site or FACS enrichment should be used in such assay.

4. Figure 4: It was clear that the target site of the TagTth-hpRNA system contained the mutation. Is it the same for siRNA? Was the siRNA designed to target the mutation? No siRNA sequence information was found in the manuscript. If the mutation was not the target site of siRNA, the siRNA control was useless in this experiment.

5. Figure 5: The AAV-TagTtH-NT control should be included to exclude the effect of injection procedure, AAV virus particle and other factors. This was an essential control.

6. Figure 6: Combination therapy is a good choice for many diseases, but not for all. The data showed that AAV-TagTtH-T-APOE2 performed better than TagTtH-T. How about APOE2 alone? Was AAV-TagTtH-T-APOE2 better than APOE2 alone? Or were they equally effective? If AAV-TagTtH-T-APOE2 was not better than APOE2 alone, there was no need for combination therapy. The control AAV-APOE2 alone was missing.

Minor points:

1. What was the function of "GAAACACCG" sequence in the U6-driven hpRNA plasmid? "gagggcctatttcccatgattccttcatatttgcatatacgatacaaggctgttagagagataattggaattaatttgactgtaaacacaaagatattagtacaaaatacgtgacgtagaaagtaataatttcttgggtagtttgcagttttaaaattatgttttaaaatggactatcatatgcttaccgtaacttgaaagtatttcgatttcttggctttatatatcttgtggaaaggacGAAACACCGNNNNNNNNNNNNNNNNNNNNAAAGTCGGCCGAAAGGCCGACTTTTTTAGCT"

2. In Figure1A, what is the functional role of each domain? Please label the functions of each domain.

3. In Fig.4E，why were the protein levels of APPwt not equal between the NT targeting groups.

4. In Figure 6 G-K, please label the X-axis groups.

**Reviewer 2**

**Were you able to assess all statistics in the manuscript, including the appropriateness of statistical tests used?**

Yes.

**Were you able to directly test the methods?**

Yes.

**Comments to author:**

This study introduces a potentially impactful approach in pathogenic allele silencing, proposing a novel TaqTth-hpRNA system aimed at targeting specific mRNA for knockdown, with particular attention to addressing the APPswe mutation implicated in Familial Alzheimer's Disease (FAD). This approach seeks to navigate the challenging terrain of providing specificity in RNA knockdown, to mitigate off-target effects that could exacerbate disease pathology.

Detailed Comments:

1.More detailed insight into how the TaqTth-hpRNA system works would enhance the abstract. A brief explanation of how it achieves the high specificity and avoids off-target effects, even in a succinct manner, would be insightful.

2. The description about hpRNA design demands further explanation and discussion regarding its feasibility. More elaboration is needed on why this particular design is considered viable and whether potential off-target effects have been contemplated. Additional theoretical or prior research is needed to support the assumptions about the interaction between TaqTth and hpRNA and its RNA helicase activity. Additional exploration into the mechanism of action of the TaqTth-hpRNA tool is warranted. In-depth insight into how the TaqTth and hpRNA interact at a molecular level to cleave target mRNA could provide valuable data and strengthen the findings.

3. The manuscript could discuss potential applications and limitations of the TaqTth-hpRNA system in a broader biological or therapeutic context. Specific scenarios where this tool may be particularly useful or faced with challenges should be examined.

4. More insight into the possible off-target effects and an analysis of cell viability post-transfection should be incorporated to gauge the safety and utility of the tool in both research and potential therapeutic applications. While the lack of sequence preference might be an advantage, further investigations into whether this leads to any unintended off-target effects or variations in efficiency depending on the mRNA sequence would add a layer of depth to the findings.

5. Consider discussing the reproducibility of the results across different experiments and, if possible, integrating experiments across various cell types to display the robustness of the TaqTth-hpRNA system.

6. Summarize findings, limitations, and future directions, framing the results in a wider context and highlighting potential steps for future research and application of the TaqTth-hpRNA system.

**Authors Response**

**Point-by-point responses to the reviewers’ comments:**

**Response to Comments of Referee #1**

Major Q1: Figure 1 showed the idea of the design of the TagTth-hpRNA. Based on reference 5 (DOI: 10.1074/jbc.M002268200), the authors created the TagTth-hpRNA system. However, no *in vitro* characterization experiments were performed. Did the tool perform as expected?

*Response:* *We thank the reviewer for the instructive suggestion on our manuscript. As you suggested, we are aware of this problem and have added the in vitro characterization in the revision. As results in revised Fig. 1 showed, the purified TaqTth protein guided by the hpRNA successfully catalyzes the cleavage of RNA targets.*

a) What was the cutting site? What were the critical amino acid residues for the cutting? Mutation at these critical residues could create "dead" mutant, which was an essential control for core experiments, in addition to the NT control.

*Response:* *(1) To identify the cutting sites, we labeled the 5'-end and 3'-end of targeted ssRNA with FAM, respectively, and designed three hpRNAs locating different positions shifted from 3' to 5' in sequence (please see in revised Fig. 1F). It was observed that the cleavage products of the 5'-end labeled ssRNA decreased from large to small (about 20-nt, 14-nt, 11-nt in sequence), while the cleavage products of the 3'-end labeled ssRNA increased from small to large (about 19-nt, 25-nt, 28-nt in sequence). As a result, we inferred that the cutting site falls near the 5' end of the probe-target duplexes region (please see in revised Fig. 1F). (2) According to the literature (Ma et al., 2000, PMID: 10827184; Shen et al., 1997, PMID: 9241249; Xu et al., 2018, PMID: 30295841), the activity of the chimeric TaqTth is influenced by the D144, K418, and Q507 sites. The 'dead' mutated dTaqTth protein was obtained by replacing these three sites with Alanine. In vitro tests proved that the dTaqTth was incapable of cleavage against RNA substrates, while the TaqTth worked well (please see in revised Fig. 1E). It will be utilized as a control in the next cellar tests as the reviewer suggested, for example in revised Fig. 2B and S7. Thanks to the reviewer for the valuable advice to make our article more convincing.*

b) What was the design principle for the Stem-Loop on the hpRNA (the content or the length of the stem or the loop)? What is the basis for selecting the Stem-Loop version used in the manuscript? Did the length of the guide sequence affect RNA cleavage? What was the optimal length?

*Response: Thank you for the referee’s helpful suggestion. (1) Since the TaqTth has a similar domain as aFEN1, we designed the basis structure of the hpRNA by imitating the hpDNA from the previously reported HpSGN (Tian et al., 2020, PMID: 33051689). As shown in revised Fig. 1D, the hp-st1 was the original version of hpRNA. Considering that hpRNA is mediated by the U6 promoter in cells, we optimized the sequence (hp-st2 version) to make the transcription able to stop at the end of the stem-loop but not terminate in the middle. We did the gel-experiment to make sure this stem-loop works well (please see in revised Fig. 1D). (2) The guide sequences of 10, 15, 20 and 25-nt length in the hpRNA were tried, respectively. Despite the fact that the 10-nt guide sequence did not work, the 15-nt, 20-nt, and 25-nt guide sequences performed well (please see it in revised Fig. 1D). We assume that the guide sequence should be long enough to bind firmly on the substrate. Hence a more than 20-nt guide sequence and the hp-st2 version of stem-loop were employed in the hpRNA.*

c) The authors did similar experiments in their previous articles that reported the development of other new editing tools (Nucleic Acids Res 2020, Genome Biol 2016), why such experiments were missing in the current work, even the TagTth-hpRNA tool were claimed better than the precious ones by the authors themselves?

*Response: The reasons we didn't perform the in vitro gel-experiments in the original article were: (1) This TaqTth-hpRNA is an RNA-mediated RNA substrate cleavage tool. In the SDS-PAGE experiment, the RNA probe and substrates are much easier to degrade than the DNA probe in our previously reported HpSGN (a DNA probe-mediated gene editing tool). The experimental operation is much more difficult. (2) Given that the TaqTth-hpRNA would eventually work in cells and animals, we ignored the in vitro characterization part. However, as you suggested, we are aware of this problem and have added an in vitro characterization into the revision. Thanks to the reviewer for the valuable advice to make our articles of higher quality. We hope the referee is satisfied with the editions we made.*

Major Q2. Figure 2 showed "the feasibility of cleaving mRNA guided by TaqTth-hpRNA in mammalian cells". However, without any in vitro or in vivo "cleavage" assays, it was hard to claim RNA "cleavage" (cleavage dead mutant was a critical control to draw such a conclusion).

a) The EGFP reporter was transiently co-transfected with the TagTth-hpRNA system, and it is well known that many factors would affect the performance of transiently transfected reporter, an EGFP stable cell line or knock-in cell line would lead to more reliable data. And even on the stable reporter cell line, the plasmids encoding TaqTth and hpRNA should be traced by different colors of fluorescent proteins. FACS analyses should gated according to these colors. In addition, as for the FACS data in Figure 2B and 2C, the EGFP-negative and the EGFP-positive populations were not clearly displayed.

*Response:* *Thank you for the referee’s valuable comments. As you suggested, we replaced the EGFP assays with an EGFP-stable HEK293T cell line (please see in revised Fig. 2B, 3G, S6 and S9), and found that the knock-down efficiencies of EGFP expression levels in the groups transfected with hpRNAs for T1, T2, T3, T4 and T5 loci were 13%, 11%, 0%, 4% and 26%, respectively. Additionally, the graphs of results with the EGFP-negative and the EGFP-positive populations were added. Since our flow cytometry can only detect two kinds of fluorescent signals, we can not trace the TaqTth and hpRNA by different colors of fluorescent proteins in this EGFP assay. Instead, we encode the TaqTth and hpRNA into a single plasmid with a mcherry tag to trace. As shown in Fig. S6, by gating according to the PE gate, the knock-down efficiencies were 20%, 16%, 12%, 19% and 30%, respectively. It* *proved that the reviewer is correct that many factors affect the performance of transiently transfected reporter. Thanks to the reviewer for the valuable advice to make our articles of higher quality.*

b) The target site for kras mRNA T6 was "5'-AAAAAUGCAUUAUAAUGUAA-3'", therefore, the DNA encoding hpRNA should be the complementary and reverse sequence "5'-TTACATTATAATGCATTTTT-3'". As we know, 5-6 Ts is the stop signal for U6 promoter-driven transcription, thus the Stem-Loop in the hpRNA could not be transcripted. However, KRAS T6 showed 56% knockdown efficiency. If this hold true, how did the TagTth-hpRNA work? This observation again required more detailed in vitro characterization of this system.

*Response:* *We repeat the KRAS experiment (with dTaqTth control) on this controversial T6 locus. As shown in Fig. S7, the knockdown efficiency of TaqTth at this locus was 54% (the data was 56% in the original manuscript), whereas dTaqTth did not produce a knockdown effect. Then we consulted literature (Arimbasseri et al., 2013, PMID: 23099421), which claims that the T cluster flanked by A is inefficient as a terminator compared to the T cluster surrounded by GC-rich sequences. We speculate that the TaqTth-hpRNA is still functional at this locus because both ends of the T cluster here are surrounded by A (on the guide sequence) and AAA (on the stem-loop sequence), respectively, which reduced its termination efficiency and made the hpRNA partly successfully transcribed. However, as the reviewer suggested, it would be better to avoid choosing loci containing A cluster. Thank you for the referee’s helpful suggestion to make our study* *more rigorous.*

c) Similar to CRISPR system, the authors also tried to determine whether the TagTth-hpRNA system required a PAM sequence as shown in Figure 2G. However, the number of target sequence was too small to draw a conclusion. Generally, a library contains sufficient sequence should be edited by the TagTth-hpRNA system in vitro or in E.coli, and deep-seq was performed before and after editing, the depleted sequences were the edited ones and PAM could be discovered in these sequences. Please refer to the articles from Dr. Jennifer Doudna or Dr. Feng Zhang or other scientists.

*Response: Thank you for the referee’s valuable comments. To clarify whether the potential preference of sequences is required for the TaqTth-hpRNA tool, referring to the CRISPR article (Cox et al., 2017, PMID: 29070703), a plasmids-library for preference screens was designed by inserting two 5’ randomized nucleotides and four 3’ randomized nucleotides (corresponding to the position of PFS in CRISPR/Cas13) separated by a target site immediately downstream of the start codon of the ampicillin resistance gene bla. To assess preferences, the positions containing randomized nucleotides in the TaqTth-hpRNA treated group were extracted and sequenced relative to the control group. No sequence preference was found in these 6 loci by sequencing analysis (please see in revised Fig. 2D). Thanks to the reviewer for the valuable advice to make our articles of higher quality.*

Major Q3: Figure 3: since TagTth-hpRNA system was more similar to CRISPR/Cas13 than siRNA, the comparison between TagTth-hpRNA and Cas13 should be shown. In Figure 3D and 3F, the inhibition of PP1B was less than 20%, which mean that there was only very weak inhibition even on the target gene. Therefore, it is reasonable that no off-target effect was observed. A more effective target site or FACS enrichment should be used in such assay.

*Response: Thanks to the reviewer for the valuable advice. We chose the classical siRNA as the comparison because it has no PFS sequence requirement and can be used for any locus as our TaqTth-hpRNA did. But the reviewer's suggestion is right. As a newly reported RNA knockdown tool, the comparison with CRISPR-Cas13 can't be ignored. Therefore, in the revised manuscript the data compared with Cas13a was added. As you can see in revised Fig. 2B and 3B,C,E, we compared the knockdown efficiency of TaqTth-hpRNA with CRISPR-Cas13a. Generally, they have the similar inhibition efficiency of EGFP and KRAS. We also add transcriptome sequencing data of HEK293T cells treated with Cas13a targeting KRAS site and looked into the cell toxicity and cell state. These added results made our study of higher quality and more convincing.*

Major Q4: Figure 4: It was clear that the target site of the TagTth-hpRNA system contained the mutation. Is it the same for siRNA? Was the siRNA designed to target the mutation? No siRNA sequence information was found in the manuscript. If the mutation was not the target site of siRNA, the siRNA control was useless in this experiment.

*Response: Thank you for pointing this out, and we sincerely apologize for our negligence in missing the siRNA sequence in the supplementary information. We have added the siRNA sequence in the revised Table S4. The siRNA used in this study had with same target sites as hpRNA.*

Major Q5: The AAV-TagTtH-NT control should be included to exclude the effect of injection procedure, AAV virus particle and other factors. This was an essential control.

*Response: Thank you for your comments. In original experiments, the AAV-TaqTth-NT control has been included* *in parallel with the non-injection control and the AAV-TaqTth-T injection group, and no significant differences were observed in behavioral and pathological aspects between the AAV-TaqTth-NT control and the non-injection group. Initially, we adopted a comparison method (Duan et al., 2022, PMID: 34312508), believing that demonstrating the comparison between the disease model mice and the treatment group alone was sufficient to illustrate the therapeutic improvement. In this revised version, following your suggestion, we have included the data for the AAV-TaqTth-NT control group and conducted statistical analysis along with the other groups (please see in revised Fig. 5).*

Major Q6: Figure 6: Combination therapy is a good choice for many diseases, but not for all. The data showed that AAV-TagTtH-T-APOE2 performed better than TagTtH-T. How about APOE2 alone? Was AAV-TagTtH-T-APOE2 better than APOE2 alone? Or were they equally effective? If AAV-TagTtH-T-APOE2 was not better than APOE2 alone, there was no need for combination therapy. The control AAV-APOE2 alone was missing.

*Response: Thanks to the reviewer for kindly pointing out that the control AAV-APOE2 alone was missing. We agree that it is essential to demonstrate the efficacy of AAV-TaqTth-T-APOE2 combination therapy. In the revised manuscript we have added it. An AAV vector expressing solely APOE2 was designated AAV-APOE2, and we conducted a comparative analysis of pathological indicators following the administration of AAV-APOE2 and AAV-TaqTth-T-APOE2 in 5×FAD mice (please see in Fig. S12). We hope the referee is satisfied with the editions we made in the current version of the manuscript.*

Minor Q1: What was the function of "GAAACACCG" sequence in the U6-driven hpRNA plasmid?

"gagggcctatttcccatgattccttcatatttgcatatacgatacaaggctgttagagagataattggaattaatttgactgtaaacacaaagatattagtacaaaatacgtgacgtagaaagtaataatttcttgggtagtttgcagttttaaaattatgttttaaaatggactatcatatgcttaccgtaacttgaaagtatttcgatttcttggctttatatatcttgtggaaaggacGAAACACCGNNNNNNNNNNNNNNNNNNNNAAAGTCGGCCGAAAGGCCGACTTTTTTAGCT"

*Response: Thanks to the reviewer for the careful review. The "GAAACACCG" sequence is the cleavage site of BbsI restriction endonuclease (GAAA/CACCCG). We use it and another restriction endonuclease SacI (AGCT/C) to replace the guide sequence for different targeted loci. As you suggested, we have expressed this part more clearly in the revised manuscript (please see in Fig. S2).*

Minor Q2: In Figure1A, what is the functional role of each domain? Please label the functions of each domain.

*Response: As the referee suggested, we added functional roles for each domain in revised Fig. 1A. By consulting the literature (Ma et al., 2000, PMID: 10827184), the C-terminal two-thirds of the proteins (297-832aa) comprises the polymerase domain, whereas the N-terminal one-third (1-297 aa) contains the 5’ nuclease domain.*

Minor Q3. In Fig.4E, why were the protein levels of APPwt not equal between the NT targeting groups.

*Response: Thanks to the reviewer for the helpful suggestion. The exposure intensity may affect the electrophoretic band's intensity. And the experiments in Fig.4E were conducted in two HEK293T cell lines stably expressing APP^swe^ or APP^wt^ (HEK293T-APP^swe^ / HEK293T-APP^wt^ cells), respectively, We have repeated the experiment (please see revised Fig. 4E) and hope to meet the expectations.*

Minor Q4: In Figure 6 G-K, please label the X-axis groups.

*Response: Thanks to the reviewer for the careful review. We have labeled the X-axis groups according to your advice (please see revised Fig. 6G-K).*

**Response to Comments of Referee #2**

Q1. More detailed insight into how the TaqTth-hpRNA system works would enhance the abstract. A brief explanation of how it achieves the high specificity and avoids off-target effects, even in a succinct manner, would be insightful.

*Response: Thanks to the reviewer for the kind suggestion. Due to the limitation of 100 words, we present a brief statement of how it achieves high specificity and efficiency in the revised abstract. It was reported (Ma et al., 2000, PMID: 10827184) that the amino acids between 331 and 596 from TthPol are responsible for the high ribonuclease activity. The specificity may inherent characteristic of the 1-328 aa of TaqPol, which contains very similar functional domains as FEN1 (Shen et al., 1996, 1997, PMID: 8621570, 9241249) inferring that polymerase may also be sensitive to mismatches on targeted substrates just as FEN1* *(Algasaier et al., 2016, PMID: 26884332). The rearrangement of these crucial structural domains enables the chimeric TaqTth to possess high efficiency and specificity. We hope the referee is satisfied with the editions we made in the current version of the abstract.*

Q2. The description about hpRNA design demands further explanation and discussion regarding its feasibility. More elaboration is needed on why this particular design is considered viable and whether potential off-target effects have been contemplated. Additional theoretical or prior research is needed to support the assumptions about the interaction between TaqTth and hpRNA and its RNA helicase activity. Additional exploration into the mechanism of action of the TaqTth-hpRNA tool is warranted. In-depth insight into how the TaqTth and hpRNA interact at a molecular level to cleave target mRNA could provide valuable data and strengthen the findings.

*Response: Thanks to the reviewer for the helpful comments. We are sorry not to illustrate the design of hpRNA clearly in detail. As the referee suggested, we added more descriptions and in vitro experiments in the revised manuscript.*

*Firstly, about the design of hpRNA. (1) Since the TaqTth has a similar domain as aFEN1, we designed the basis structure of the hpRNA by imitating the hpDNA from the previously reported HpSGN (Tian et al., 2020, PMID: 33051689). As shown in revised Fig. 1D, the hp-st1 was the original version of hpRNA. Considering that hpRNA is mediated by the U6 promoter in cells, we optimized the sequence to make the transcription able to stop at the end of the stem-loop but not terminate in the middle (hp-st2 version). We did the gel-experiment to make sure this stem-loop works well (please see in revised Fig. 1D). (2) The guide sequences of 10, 15, 20 and 25-nt length in the hpRNA were tried, respectively. Despite the fact that the 10-nt guide sequence did not work, the 15-nt, 20-nt, and 25-nt guide sequences performed well (please see it in revised Fig. 1D). We assume that the guide sequence should be long enough to bind firmly on the substrate. Hence a more than 20-nt guide sequence and the hp-st2 version of stem-loop were employed in the hpRNA.*

*Next, about the TaqTth and hpRNA interaction: (1) According to the literature (Ma et al., 2000, PMID: 10827184; Shen et al., 1997, PMID: 9241249; Xu et al., 2018, PMID: 30295841), the activity of the chimeric TaqTth is influenced by the D144, K418, and Q507 sites. The 'dead' mutated dTaqTth protein was obtained by replacing these three sites with Alanine. In vitro tests proved that the dTaqTth was incapable of cleavage against RNA substrates, while the TaqTth worked well (please see in revised Fig. 1E). It will be utilized as a control in the next cellar tests. (2) As the referee suggested, we proved the TaqTth and dTaqTth could combine with hpRNA at the molecular level by EMSA (see in revised Fig. S1). But we did not explore the RNA helicase activity since target mRNA generally exists in single-stranded form. (3) To further identify the cutting sites, we labeled the 5'-end and 3'-end of targeted ssRNA with FAM, respectively, and designed three hpRNAs locating different positions shifted from 3' to 5' in sequence. It was observed that the cleavage products of the 5'-end labeled ssRNA decreased from large to small (about 20-nt, 14-nt, 11-nt in sequence), while the cleavage products of the 3'-end labeled ssRNA increased from small to large (about 19-nt, 25-nt, 28-nt in sequence). As a result, we inferred that the cutting site falls near the 5' end of the probe-target duplex region (please see in revised Fig. 1F).*

*Thanks to the reviewer for the valuable advice to us, making our article of higher quality and more convincing, and hope to meet the expectations.*

Q3. The manuscript could discuss potential applications and limitations of the TaqTth-hpRNA system in a broader biological or therapeutic context. Specific scenarios where this tool may be particularly useful or faced with challenges should be examined.

*Response: Thanks to the reviewer for the valuable advice. Compared with the robust CRISPR-associated systems, the TaqTth-hpRNA is just a potential choice at present. More work is still needed to further optimize the efficiency by testing more protein variants and hpRNA variants. Then as an RNA cleaving tool, TaqTth-hpRNA cannot achieve RNA base replacement. In addition, there is still concern about the AAV delivery’s potential safety risks. Based on the animal-level APP^swe^ tests by a single AAV combined with APOE2, the TaqTth-hpRNA could be used to inhibit pathogenic mRNA for gene therapy. The successful and efficient cleavage described in this study also encouraged us to investigate whether TaqTth-hpRNA can also effectively cleave bacterial RNA. Furthermore, whether TaqTth-hpRNA could be used for DNA editing is worth exploring. The compact dead TaqTth protein also provides an alternative to being modified with effectors with broad genome engineering applications, including RNA regulation and RNA imaging in future studies.*

*As the referee suggested, we added a concise context and Fig. 7 to summarize the potential application and limitations in the discussion section of the revised manuscript. We hope the referee is satisfied with the editions we made.*

Q4. More insight into the possible off-target effects and an analysis of cell viability post-transfection should be incorporated to gauge the safety and utility of the tool in both research and potential therapeutic applications. While the lack of sequence preference might be an advantage, further investigations into whether this leads to any unintended off-target effects or variations in efficiency depending on the mRNA sequence would add a layer of depth to the findings.

*Response: Thanks to the reviewers for the valuable comments. We are sorry not to illustrate the part of off-target effects in detail. As the referee suggested, we added more experiments and descriptions in the revised manuscript.*

*Firstly, we added the transcriptome sequencing analysis of siRNA, CRISPR/Cas13, and TaqTth-hpRNA system to compare their off-target effects (please see in revised Fig. 3D, E and F). We also conducted comparative visual inspections of cell states before and after transfection and analyzed cellular viability (please see in revised Fig. 3B and C). In general, TaqTth and Cas13 showed better specificity and lower cytotoxicity than siRNA.*

*Next, we analyzed the reason why the non-sequence-specific preference does not result in any unexpected off-target effects and exhibits varying editing efficiencies for different mRNA substrates. As we talked about above, the specificity may inherent characteristic of the N-terminal of TaqPol, which contains very similar functional domains as FEN1 (Shen et al., 1996, 1997, PMID: 8621570, 9241249) inferring that polymerase may also be sensitive to mismatches on targeted substrates just as FEN1 (Algasaier et al., 2016, PMID: 26884332). The rearrangement of these crucial structural domains enables the chimeric TaqTth to possess high specificity. It is somewhat analogous to the varying cleavage efficiencies of siRNA on different targets. The binding of hpRNA with the targeted RNA is influenced by the secondary structure of the targeted RNA itself. And a high GC content may result in high melting temperature (Tm) values, leading to a more stable and higher-affinity binding of hpRNA with the targeted RNA.*

*As the referee suggested, we added the analysis and discussion in the revised manuscript. Thanks to the reviewer for the valuable advice to make our article more convincing.* *We hope the referee is satisfied with the editions we made.*

Q5. Consider discussing the reproducibility of the results across different experiments and, if possible, integrating experiments across various cell types to display the robustness of the TaqTth-hpRNA system.

*Response: We thank the reviewer for the helpful comments. Following the suggestion, we added validation on the TaqTth-hpRNA system in different cell lines, and the results are shown in revised Fig. 4F . We hope the referee is satisfied with the editions we made in the current version of the manuscript.*

Q6. Summarize findings, limitations, and future directions, framing the results in a wider context and highlighting potential steps for future research and application of the TaqTth-hpRNA system.

*Response: As the referee suggested, we added an overview figure (please see in added Fig. 7) and a discussion part to summarize the findings, limitations, and future directions in the revised manuscript. Thanks to the reviewer for the valuable advice to make our articles of higher quality. We hope the referee is satisfied with the editions we made.*

**Second round of review**

**Reviewer 1**

The authors have addressed most of my concerns.

**Reviewer 2**

The manuscript reflects careful consideration of reviewer feedback, with detailed responses and additional experiments to address the concerns raised, enhancing the credibility of the findings.

While the manuscript provides some insights into the mechanism of action of TaqTth-hpRNA, further exploration into the molecular interactions between TaqTth and hpRNA could strengthen the findings. Detailed structural studies or molecular dynamics simulations could be beneficial.

There are minor typographical errors throughout the manuscript that should be corrected to improve readability.

**Authors Response**

**Point-by-point responses to the reviewers’ comments:**

Referee #2

Q1: The manuscript reflects careful consideration of reviewer feedback, with detailed responses and additional experiments to address the concerns raised, enhancing the credibility of the findings. While the manuscript provides some insights into the mechanism of action of TaqTth-hpRNA, further exploration into the molecular interactions between TaqTth and hpRNA could strengthen the findings. Detailed structural studies or molecular dynamics simulations could be beneficial.

*Response: Thanks to the reviewer for the valuable advice. We try to follow your request to explore the molecular structure of TaqTth protein and its dynamics. Although we do not have Cryo-SEM, we used AlphaFold Server to predict the molecular interaction between TaqTth and hpRNA, shown in Additional file 1: Fig. S1A, including TaqTth, hpRNA, targeted RNA, and two Mg2+. The Ramachandran Plots and Sequence coverage images show good confidence. So we think this simulation has a high confidence level. In the future, we will create conditions to explore the structure of the protein, such as the key amino acids that bind to hpRNA and target, and the actual molecular conformation of the protein. Thank you for your positive comments on the manuscript. We hope the referee is satisfied with the editions we made.*

Q2: There are minor typographical errors throughout the manuscript that should be corrected to improve readability.

*Response: Thank you for kindly pointing the mistakes out. We have carefully checked and revised the manuscript. We hope the referee is satisfied with the editions we made.*
